# In target areas where human mosquito-borne diseases are diagnosed, the inclusion of the pre-adult mosquito aquatic niches parameters will improve the integrated mosquito control program

**DOI:** 10.1371/journal.pntd.0008605

**Published:** 2020-08-14

**Authors:** Swapan Kumar Ghosh, Dipanjan Podder, Atanu Kumar Panja, Sabyasachi Mukherjee

**Affiliations:** Molecular mycopathology lab, Biological Control and Cancer Research Unit, PG Department of Botany, Ramakrishna Mission Vivekananda Centenary College (Autonomous), Rahara, Kolkata, WB, India; Faculty of Science, Mahidol University, THAILAND

## Abstract

In human communities inhabiting areas–such as West Bengal- India–where perpetuate the pre-imago & adult developmental stages of mosquitoes; many infectious diseases are still diagnosed such as Dengue, Malaria and Acute Encephalitis Syndrome. The control of the aquatic developmental stages is one of the easiest way to prevent the emergence of adults—the blood feeding adult females being thus prevented to sample their blood meal and to lay their eggs in the aquatic milieu where develop the aquatic pre-imaginal developmental stages. Moreover, reducing the adult population size also the probability of for the blood feeding adult female mosquitoes to act as hosts and vectors of the arboviruses such as dengue virus & Japanese encephalitis virus as well as of *Plasmodium*. Several environmental factors including water quality parameters are responsible for the selection of oviposition sites by the female mosquitoes. In our study, larval densities of three important mosquitoes (*Aedes*/*A*. *albopictus*, *Anopheles*/*An*. *stephensi* and *Culex*/*C*. *vishnui*) were measured and water qualities of their habitat i.e. pH, Specific Conductance, Dissolved Oxygen, Chemical Oxygen Demand, Total alkalinity (T_alk_), Hardness, Nitrate nitrogen and Ammonia nitrogen were analyzed in 2017 and 2018 in many districts of West Bengal where humans beings are suffering from arboviruses and /or malaria. Whereas we have found positive correlation of density of C. *vishnui* and *A*. *albopictus* with the water factors except Chemical Oxygen Demand (COD) and T_alk_, for *An*. *stephensi* all these factors except pH, COD and T_alk_ have positive correlation. Hardness of the water shows positive correlation with the density of *An*. *stephensi* and *C*. *vishnui* but negative correlation with density of *A*. *albopictus*. Contour plot analysis demonstrates that occurrence of each mosquito species lies in between specific range of water factors. Inter- correlation analysis revealed that mosquitoes were negatively correlated with each other. A positive correlation of the water quality parameters and larval density, over two successive years, was also noticed. In conclusion, the increasing level of pollution due to industrial and other irresponsible waste management system which changes the water quality parameters may also influence mosquito population.

## Author summary

West Bengal has been battling against several mosquito borne diseases such as Malaria, Dengue and Acute Encephalitis Syndrome (AES) in recent years. In our study, mosquito larval density of three mosquito species (*An*. *stephensi*, *A*. *albopictus* and *C*.*vishnui*) was measured in the years 2017 and 2018, where most of the mosquito- borne disease cases were reported in West Bengal. Correlation of larval density of three mosquito species with habitat water factors (pH, Hardness, Dissolved Oxygen, Ammonia nitrogen etc.) was determined. Inter- correlation of three different mosquito species was also analyzed. The results demonstrate mosquito larval density of each species were correlated negatively or positively with different water factors and their occurrence lies in between specific range of different water factors. Mosquitoes exhibited negative inter- correlation with each other. Larval density was found to be increased in most of the spots in 2^nd^ sampling year. We may interpret that, increased pollution of water during the year, which changes water quality, is a positive enhancer of mosquito population. Therefore, determining water quality parameters are most important to design the mosquito eradication program in target areas.

## Introduction

Mosquito borne diseases are becoming a threat to public health as malaria, dengue and encephalitis are increasing rapidly in the society and posing as hazardous disease that can take many people’s lives if they are not eradicated properly. Mosquito borne diseases infect more than 7×10^6^ people around the world each year whereas, 2×10^6^ people end up dying annually [1]. In recent years, West Bengal is facing severe human health concerns due to mosquito borne diseases. According to the report of Directorate of Health Service (DHS), Govt. of West Bengal, 35236 malaria cases, 22685 dengue cases and 2004 acute encephalitis syndrome (AES) were reported with 59, 45 and 275 deaths respectively in 2016 [2]. One of the most important parts of the mosquito life cycle is larval aquatic stage. Aquatic habitat of mosquito possesses a wide range of physicochemical factors that may strongly influence the abundance of different mosquito involving disease transmission. Many environmental factors may have effect on larval distribution, density, survivability and development. Nowadays, physico-chemical nature of water is changing due to industrial and other irresponsible waste management system which contaminates ground water, and acid rain which is the result of increased environmental pollution.

The physico-chemical nature of water might act as a determining factor for the selection of oviposition and breeding sites of mosquitoes[3]. It was reported that water quality determines whether female mosquitoes will lay eggs or the immature stages of mosquitoes develop into the adult stage[4]. Although, diagnostic and scientific research has showed that habitats without Oxygen tension are preferred by many mosquito species, some of them breed in open pools where the area is fully exposed to sunlight [5].Hagstrum and Gunstream [6] have identified two groups of mosquito species: one of the groups have a limited abundance and the other have a widespread abundance in relation to inorganic ions. Suman *et al*.[7] reported in 2014 that the presence of oxygen in water was a positive sign for the growth of several mosquito species.

In our present research, for mosquito’s larval habitat study we have selected those districts of West Bengal which were most affected by mosquito borne diseases in 2016. According to the report of DHS, WB, most malaria cases were reported from Puruliya (3305 cases) and Kolkata Municipal Corporation (KMC) areas (16498 cases), most dengue cases were reported from North 24 Parganas (8250 cases) and Haora (2237 cases) and most Acute Encephalitis Syndrome (AES) cases were reported from Bardhamman (145 cases) and Alipurduar (169 cases) in 2016 [2]. *Anopheles stephensi* is considered to be the primary vector for transmitting malaria in urban areas asreported by National vector borne disease control programme which was conducted in 2014–15 by Directorate General of Health Services (DGHS), Ministry of health & family welfare, Government of India. Similarly, *Culex vishnui* is reported to be the primary vector for transmitting encephalitis and *Aedes albopictus* is found to be responsible for transmitting Dengue (DGHS, India, 2014–2015) [8]. With the help of local health workers we have identified some areas of the afore mentioned districts where the vectors (mosquito larvae) of malaria, dengue and encephalitis were found to occur in abundance. Mosquito larvae were identified. Larval densities of three medicinally important mosquitoes were measured and water quality factors of each habitat were analyzed. How these factors affect larval density in different habitats in the case of three mosquitoes was carefully examined and analyzed. Therefore, the primary objective of this research work is to correlate water quality parameters with larval density in the select two years, and also make an analysis based on the findings of careful examination about three mosquito’s inter-correlation with water quality parameters. A thorough knowledge of the physico-chemical factors which influence mosquito habitat on larval production and a good understanding of the biological and ecological aspects of mosquito vector species are of great importance in the case of formulating effective plan and careful implementation of integrated vector control strategies by environmental management. Based on our laborious and assiduous reading about the existing research in this particular field we wish to bring to the notice with emphasis that it is the first attempt of any kind of research work in this field about the water quality analysis in respect to larval density of three medicinally important mosquitoes in the respective disease affected districts of West Bengal.

## Materials and methods

### Study area and identification of larvae

We have selected five disease affected districts of West Bengal, India by literature study (S1 Fig). Areas of KMC and Puruliya (most malaria cases reported) were surveyed for larval habitat of *An*.*stephensi*, North 24 Parganas and Haora (most dengue cases reported) were screened for larval habitat of *A*.*albopictus*,and Alipurduar and Burdwan (most AES cases reported) for *C*. *vishnui* larvae habitat study(DHS, WB, 2016).We have identified and took samples from five blocks, each with three different habitats of three respective disease spreading mosquito vectors by field study in every disease affected district(S1 Fig). All samplings were done on public lands. So, a total of ten blocks in two districts with thirty different habitats for each mosquito was identified for habitat study, and these are mentioned with coordinates within brackets. Mosquito larvae were identified with the help of pictorial key [9] and confirmed by scientist of Zoological Survey of India. In KMC area, *An*. *stephensi* larval habitats were mainly found in the areas of Borough I (22.617889, 88.370556), Borough IV(22.586861, 88.351667), Borough VII (22.55525, 88.378611), Borough XI (22.491833, 88.383694) and Borough XV (22.481833, 88.316694),and in Puruliya district, *An*. *stephensi* larvae were found to be occurred in the blocks of Arsha(23.3264194,86.1660509), Jhalda II(23.3028632,86.6482258), Balarampur (23.0940236,86.2139204), Puruliya I (23.3308211,86.3273684) and Puruliya II (23.4881729,86.5562797) by random field study. In North 24 Parganas, *A*. *albopictus* larval habitats were found in Basirhat(22.6601025,88.8499545), Haroa(22.6079539,88.6731112), Bangoan (23.0430912,88.8100947), Habra (22.8440812,88.6493081) and Swarupnagar (22.8117446,88.8467814) and in Haora, *A*. *albopictus* larval habitats were mainly found in the areas of Bally (22.6457334,88.3391052), Sankrail (22.5431297,88.2038402), Uluberia (22.4693578,88.0529668), Domjur (22.6254437,88.1929857) and Bagnan I (22.4643467,87.9588736)by random field study.*C*. *vishnui* larval habitats were found in Alipurduar I (26.5189189,89.4767753), Alipurduar II(26.3896047,89.7365952), Falkata (26.5193448,89.1654674), Kumargram (26.6128239,89.8074704) and Kalchini(26.68674,89.4453446) areas of Alipurduar districts and in Burdwan districts, *C*. *vishnui* larval habitats were found to be occurred in the areas of Kalna II (23.1969095,88.2715329), Galsi II(23.3447561,87.6710485), Rayna II(23.072774,87.8727204), Katwa I(23.6053212,88.1690036) and Burdwan I (23.2579754,87.9761552) by random field survey. Three larval habitats were randomly selected from each area for the study of water quality and density determination. The average temperature readings of these districts at the end of August were ranging from 24.00±2°C (Min) to 34.3±2°C (Max) with high humidity of 86%-91%. Average rainfall in rainy season was 170-313cm. At the end of the rainy season, many grassy ditches, roadside ditches, potholes created on the roadside by vehicle’s wheel tracks, blocked drains, marshy swamps, water pools in cultivation field of jute during retting, artificial container like broken sinks left at construction sites were filled with stagnant rain water, and mosquito larvae created colony as these places are ideal locations for their habitat. In the figure given below (S2 Fig), some photographs are presented of larval habitats of three mosquito species the sample of which was collected for the analysis of the examination. (S2 Fig).

### Sampling of habitat water, mosquito larvae

Larvae and water sampling from the selected larval habitats were collected from the areas of six districts during the end of rainy season in the last week of August. All samples were collected from open access public areas. At first, inspection was done for the presence of medicinally important three mosquito larvae with high density and the coordinates were recorded using a GPS unit. Three mosquito habitats were selected randomly in each area and total ten areas in two districts with thirty habitats were selected for each mosquito. So a total of ninety larval habitats for three mosquitoes were identified in mosquito borne disease affected six districts of West Bengal. For larvae sampling, soup ladle of 10 cm diameter with water holding capacity of 100 ml was used [10, 11]. The soup ladle was dipped into the water pool to collect larvae. Three samplings were taken from each habitat.

After collection water samples were preserved following the procedure mentioned in the manual of Kori *et*. *al*.[12]. Narrow mouth glass B.O.D. bottle (300ml) with DO sampler was used to collect water sample for DO analysis.B.O.D. bottles were let to overflow while collecting water samples and then stopper the bottle to avoid air bubbles formation. After that, 1ml of MnSO_4_ and 1ml of alkali-iodide-azide reagent were added by using micropipette up to the brim immediately. Then glass stopper was put immediately. The reagents were mixed up by inverting the bottles 2–4 times and allowed to settle down the precipitate. After that, 1ml of H_2_SO_4_ was added by replacing stopper and mixed well until the precipitates were properly mixed into the solution. After acidification, the samples were packed in crushed ice containing sample box before further analysis inside laboratory within 8 hrs.

For COD, Ammonia nitrogen and Hardness analysis, samples were collected in 500ml glass bottles and were preserved by acidification to pH≤2 using conc. H_2_SO_4_ and packed in crushed ice inside sample box before analysis in laboratory. For total alkalinity, Nitrate nitrogen and specific conductance analysis the samples were collected in glass container and were packed in crushed ice inside sample box for preservation before analysis in laboratory.

### Water quality analysis

Having collected and preserved the water samples by aforementioned techniques, they were taken to the laboratory for further analysis in a sample box. Physico-chemical parameters like pH, Specific Conductance (SC), Dissolved Oxygen (DO), Chemical Oxygen Demand (COD), Total alkalinity (T_alk_), Water Hardness, Ammonia nitrogen, Nitrate nitrogen of water samples which were collected from each mosquito habitat were assayed at laboratory following the manual [12]. 

pH of the water samples was measured by electrometric method using a pH meter consisting of potentiometer, a glass electrode, a reference electrode and a temperature compensating device. Specific conductance was measured using conductivity meter and due procedures were followed as per instruction manual. Dissolved Oxygen of the water sample was analysed by the Winkler method with azide modification. COD of the water samples was analyzed by open reflux method. Total alkalinity of sample was estimated by titrating with standard 0.02N H_2_SO_4_ where phenolphthalein and methyl orange were used as indicator. Water Hardness was analyzed by EDTA titration method, Ammonia nitrogen of the water samples was assayed by Nesslerisation method. Nitrate nitrogen of the water samples were measured by UV spectrophotometer method.

### Data analysis

#### Larval population estimation

The total population of larvae (P) in each habitat was calculated by the following formula. P = vn/c, where c = water holding capacity of the ladle, v = the volume of the water contained in the pool, n = mean number of larvae per dip [13].Larval population was estimated for each habitat by MSEXCEL 2007.

#### Larval density estimation

Density of mosquito larvae in a habitat was measured as the number of larvae per dip collected from respective mosquito habitat.

Density index of each mosquito species was analysed as percent of specimens in the total larval sample according to the following formulae [14].

$$LDI\;(Larval\;density\;index)=\frac{Number\;of\;larvae\;of\;one\;type(l)}{Total\;number\;of\;larvae(L)}\times 100$$

Where, *l* = number of mosquito larvae of a species, *L* = number of total larvae collected.

### Multivariate analysis of three mosquito species with different environmental variables

Environmental variables like pH, Specific conductance (SC), Dissolved Oxygen (DO), Chemical Oxygen Demands (COD), Total alkalinity, hardness, Ammonia nitrogen content (AN) and Nitrate nitrogen (NN) content of the water samples were analysed in the laboratory. Correlation between different environmental factors with larval density at different habitats of each mosquito was examined using Pearson’s product moment correlation testafter log transformation and 2D kernel test was done to interpret the optimum range of different physico-chemical factors for three mosquito species in two sampling years. Variables were plotted using ggplot2 through R (Ver: 3.4.4) i.e. Contour plot. The range of physico-chemical factors of habitats where respective mosquitoes were found were carefully observed and identified. For every water quality parameter, we have taken the highest and lowest value of the parameters, where and in between which mosquito habitat has found, and interpreted as optimum range for those parameters for respective mosquito species. Significance of the correlation was tested by Pearson’s chi square test. Multiple logistic regression equations were carried out for three mosquito species to elucidate how the water quality parameters affect the respective mosquito larval density.

### Analysis of inter- association of mosquito larvae

We have done an inter-correlation study for detecting the possible effect of inter-relation among three mosquito species. We have performed Spearman correlation analysis with R (Ver: 3.4.4) as our data was non-normally distributed and plotted with ggcorrplot2.

### Analysis of correlation of water quality parameters and larval density for each larval habitat with years

We have analysed spot- wise density data and environmental parameters of each species in the respect of years. After assembling them, we have performed Spearman correlation with R (Ver: 3.4.4) analysis for detecting the change of water quality parameters within the two sampling years at each spot.

## Results

### Total estimated population and density of mosquito larvae in study area and their identification

Mosquito larvae were identified as *Anopheles stephensi*, *Aedes albopictus* and *Culex vishnui*. In 2017, the total estimated *An*. *stephensi* larval population in our identified ten areas of two districts (KMC and Puruliya) was 5031.25. In 2018, the total estimated anopheline larvae population was increased to 5292 in those areas. Total estimated larval population of C.*vishnui* in our selected ten areas of two districts (Alipurduar& Burdwan) was 45,823.66 in 2017, and in 2018, the larval population of *C*.*vishnui* was increased to 48,383.76 in those areas. Total larval population of *A*. *albopictus* was estimated to 1343.88 in 2017 and it increased to 1455.5 in 2018 in selected ten areas of two districts (N 24 Parganas & Haora). Mosquito populations were found to be increased in most of the habitats of every disease affected districts of West Bengal in 2018 for three mosquitoes. District- wise total larvae population data are presented in the S1 Table.

**Fig 1:**
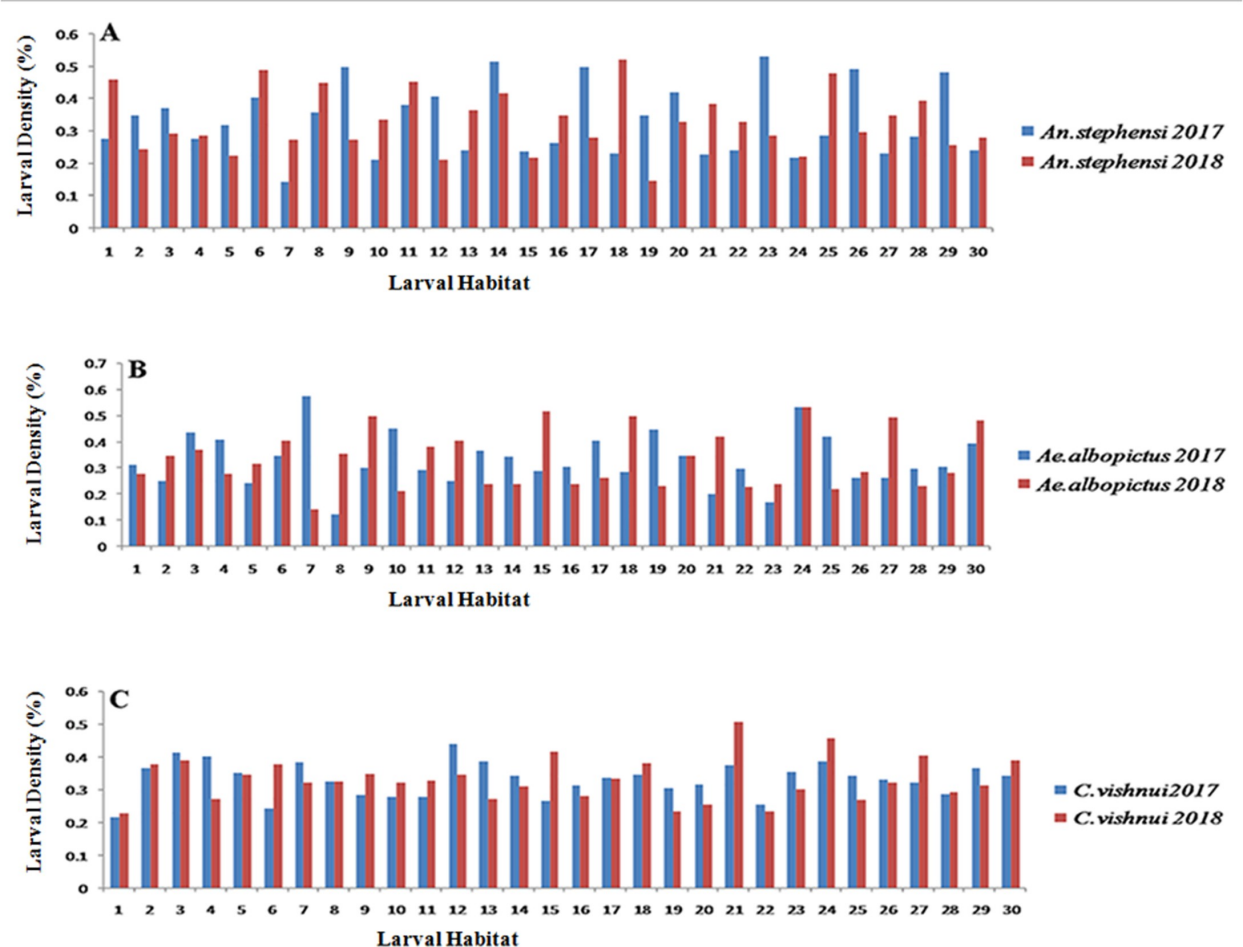
Habitat-wise density of *An*. *Stephesni*, *A*. *albopictus* and *C*. *vishnui* larvae in 2017 and 2018 in the respective districts of West Bengal.

Habitat-wise mosquito larval density in 2017 and 2018 for three mosquitoes is represented by MS Excel graphs which exhibits an increased larval population at most of the habitats in 2018 for all three mosquitoes (Fig 1).

Larval density index of three mosquito species is presented in S2 Table.

Density of each mosquito species in a single habitat to total number of larvae collected in that area during two years of sampling is calculated and presented (S3, S4 & S5 Tables).

### Association with mosquito larval density and physico-chemical factors of water

The results(Table 1) of Pearson correlation tests for water samples of 2017 reveals that larval density of *An*. *stephensi* in selected 30 larval habitats of two districts (KMC and Puruliya) are in negative correlation with pH of the water (r: -0.851, P: 2.523e-09), Chemical Oxygen Demand (r: -0.911, P:2.87e-12) and Total alkalinity (r: -0.0966, P: <2.2e-16) but larval density of *An*. *stephensi* is in positive correlation with parameters like Specific conductance (r: 0.871, P: 3.914e-10), Dissolved Oxygen (r: 0.963, P:<2.2e-16), Hardness (r: 0.919, P: 7.86e-13), Ammonia nitrogen (r: 0.971, P:<2.2e-16) and Nitrate nitrogen (r: 0.707, P: 1.251e-05).Larval density of *C*.*vishnui* in select 30 habitats of two districts (Alipurduar and Burdwan) in 2017 was found to be positively correlated with pH (r: 0.969, P: <2.2e-16), Specific conductance (r: 0.897, P: 1.921e-11), Dissolved Oxygen (r:0.971, P: < 2.2e-16), Hardness (r:0.970, P:<2.2e-16), Ammonia nitrogen (r:0.918, P: 9.068e-13)and Nitrate nitrogen (r: 0.953, P: 4.618e-16) and on the other hand it was negatively associated with COD (r: -0.932, P: 7.064e-14), Total alkalinity (r: -0.967, P:< 2.2e-16). In case of *A*.*albopictus*, larval density in select 30 habitats of two districts (North 24 Parganas and Haora) was positively associated with pH (r: 0.989, P: < 2.2e-16), Specific conductance (r: 0.868, P: 5.377e-10), Dissolved Oxygen (r: 0.747, P: 2.088e-06), Ammonia nitrogen (r: 0.838, P: 7.153e-09) and Nitrate nitrogen (r: 0.835, P: 8.984e-09) but it was negatively correlated with Chemical Oxygen Demand (r: -0.984, P:<2.2e-16), Total alkalinity (r: -0.924, P: 3.198e-13) and Hardness (r:-0.945, P: 3.226e-15).The results of correlation analysis are presented in Table 1.

**Table 1:** Pearson correlation of Physico-chemical parameters of water in relation to larval density of three mosquito species in 2017.

Mosquito larvae species	Statistical value	Water quality parameters							
		pH	SC	DO	COD	T_alk_	Hardness	AN	NN
*An*. *stephensi*	r	-0.851	0.871	0.963	-0.911	-0.966	0.919	0.971	0.707
	p	2.523e-09	3.914e-10	<2.2e-16	2.87e-12	< 2.2e-16	7.86e-13	< 2.2e-16	1.251e-05
*C*. *vishnui*	r	0.969	0.897	0.971	-0.932	-0.967	0.970	0.918	0.953
	p	<2.2e-16	1.921e-11	< 2.2e-16	7.064e-14	< 2.2e-16	< 2.2e-16	9.068e-13	4.618e-16
*A*. *albopictus*	r	0.989	0.868	0.747	-0.984	-0.924	-0.945	0.838	0.835
	P	< 2.2e-16	5.377e-10	2.088e-06	< 2.2e-16	3.198e-13	3.226e-15	7.153e-09	8.984e-09

The results (Table 2) of Pearson correlation tests for water samples of 2018 exhibited that larval density of *An*. *stephensi* was also negatively associated with pH of the water (r: -0.973, P:< 2.2e-16), Chemical Oxygen Demand (r: -0.908, P: 4.029e-12) and Total alkalinity (r: -0.899, P: 1.489e-11) but it was found to be positively associated with parameters like Specific conductance (r: 0.963, P: < 2.2e-16), Dissolved Oxygen (r: 0.955, P: 2.777e-16), Hardness (r: 0.937, P: 2.249e-14), Ammonia nitrogen (r: 0.963, P: <2.2e-16) and Nitrate nitrogen (r: 0.918, P: 8.257e-13).Larval density of *C*.*vishnui* in select 30 habitats of two districts (Alipurduar and Burdwan) in 2018 was found to be positively correlated with pH (r: 0.952, P:5.326e-16), Specific conductance (r: 0.979, P: <2.2e-16), Dissolved oxygen (r: 0.879, P: 1.62e-10), Hardness (r: 0.960, P: < 2.2e-16), Ammonia nitrogen (r: 0.953, P: 3.997e-16) and Nitrate nitrogen (r: 0.923, P: 4.084e-13) but it was negatively associated with COD (r: -0.951, P: 6.869e-16) and Total alkalinity (r: -0.938, P: 1.897e-14). Larval density of *A*. *albopictus* in select 30 habitats of two districts (North 24 Parganas and Haora) was positively associated with pH (r: 0.908, P: 4.145e-12), Specific conductance (r: 0.933, P: 5.785e-14), Dissolved Oxygen (r: 0.916, P: 1.145e-12), Ammonia nitrogen (r: 0.878, P: 1.822e-10) and Nitrate nitrogen (r: 0.922, P: 5.058e-13) but it was negatively correlated with Chemical Oxygen Demand (r: -0.918, P:<7.849e-13), Total alkalinity (r: -0.888, P: 5.497e-11) and Hardness (r: -0.945, P: 3.824e-15).The results of correlation analysis are presented in Table 2.

**Table 2:** Pearson correlation of Physico-chemical parameters of water in relation to larval density of three mosquito speciesin 2018.

Mosquito larvae	Statistical value	Water quality parameters							
		pH	SC	DO	COD	T_alk_	Hardness	AN	NN
*An*. *stephensi*	r	-0.973	0.963	0.955	-0.908	-0.899	0.937	0.963	0.918
	p	< 2.2e-16	< 2.2e-16	2.777e-16	4.029e-12	1.489e-11	2.249e-14	< 2.2e-16	8.257e-13
*C*. *vishnui*	r	0.952	0.979	0.879	-0.951	-0.938	0.960	0.953	0.923
	p	5.326e-16	<2.2e-16	1.62e-10	6.869e-16	1.897e-14	<2.2e-16	3.997e-16	4.084e-13
*A*. *albopictus*	r	0.908	0.933	0.916	-0.918	-0.888	-0.945	0.878	0.922
	P	4.145e-12	5.785e-14	1.145e-12	7.849e-13	5.497e-11	3.824e-15	1.822e-10	5.058e-13

### Optimum range analysis of different physico-chemical factors for three mosquitoes

**Fig 2:**
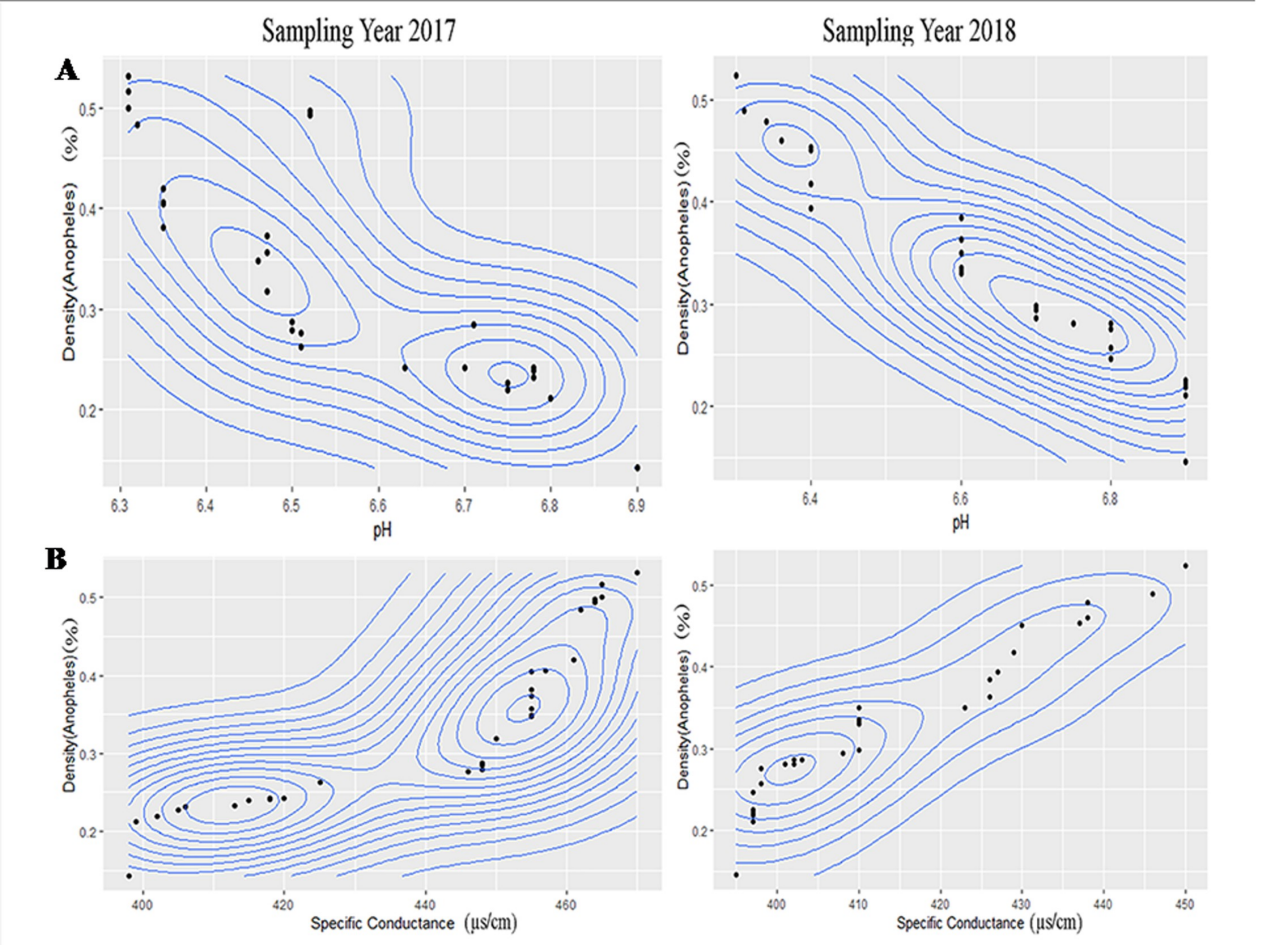
Contour plot graph exhibiting correlation of (A) pH and (B) Specific Conductance, with larval density of *An*. *stephensi*.

**Fig 3:**
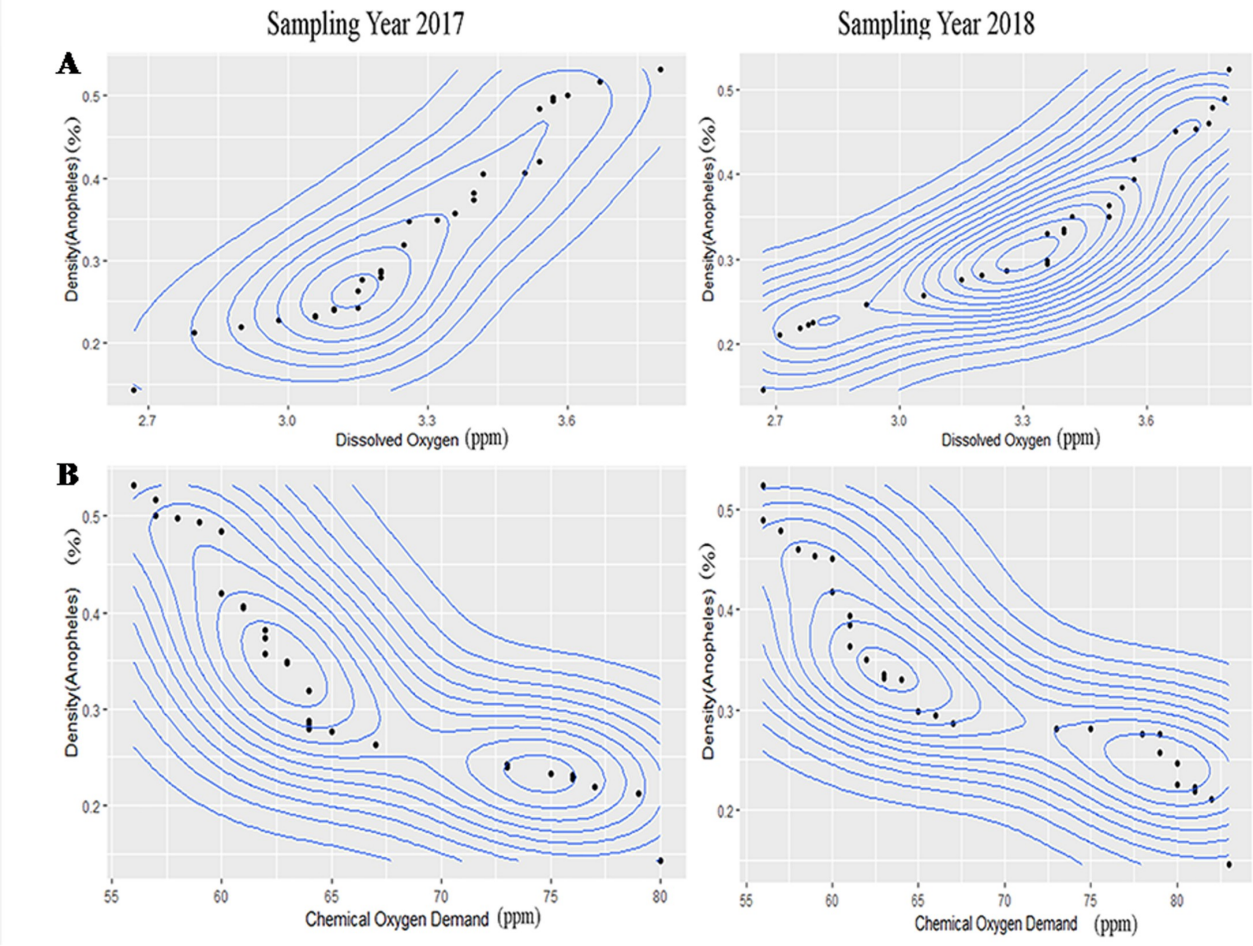
Contour plot graph exhibiting correlation of (A) DO and (B) COD with larval density of *An*. *stephensi*.

**Fig 4:**
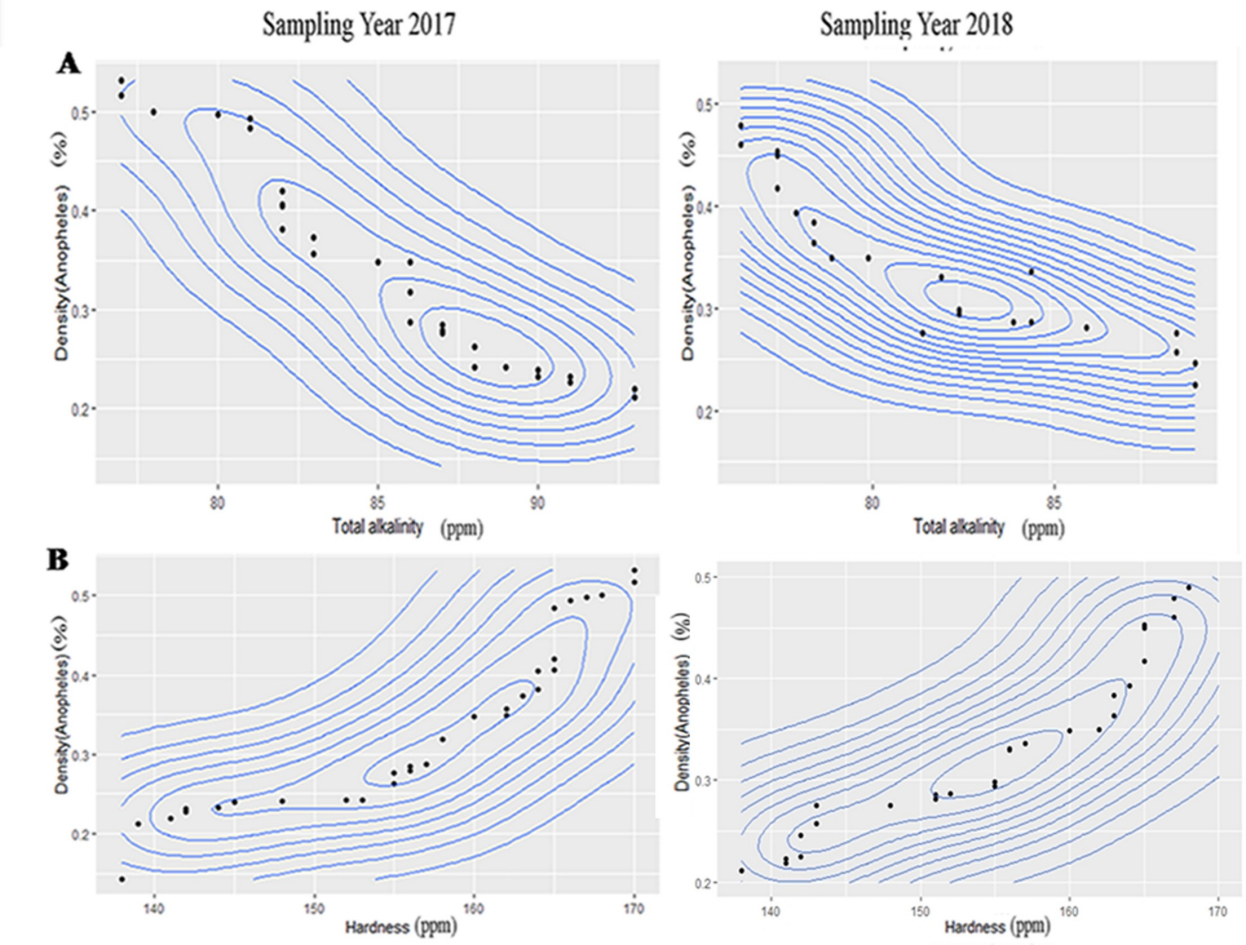
Contour plot graph exhibiting correlation of (A) Total alkalinity and (B) Hardness with larval density of *An*. *stephensi*.

**Fig 5:**
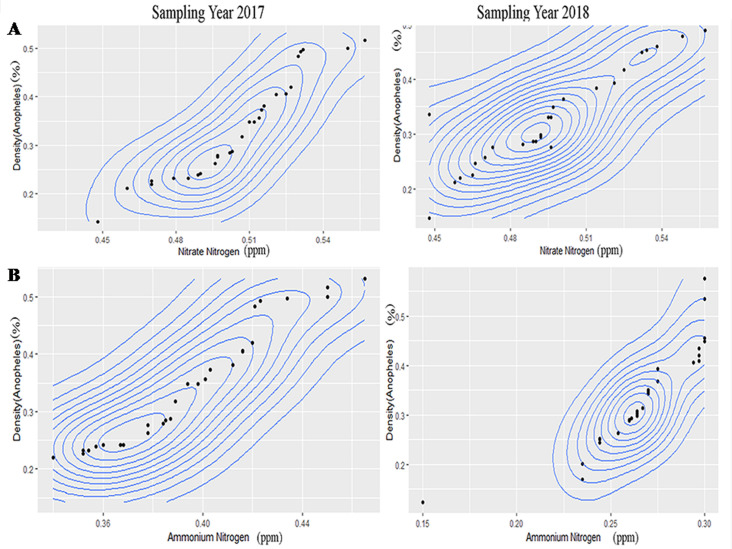
Contour plot graph exhibiting correlation of (A) Nitrate nitrogen and (B) Ammonia nitrogen with larval density of *An*. *stephensi*.

The results of contour plot analysis for sampling year 2017& 2018 exhibited that optimum range of pH for *An*. *stephensi* was 6.31–6.90 (Fig 2A) whereas in case of *A*. *albopictus* the optimum range of pH was 7.09–8 (S3A Fig) and for *C*.*vishnui* it was 7.60–8.40(S7A Fig). Optimum range of Specific conductance for habitat water of *An*. *stephensi* was 395–470μs/cm (Fig 2B), for *A*. *albopictus* it was 338–503 μs/cm(S3B Fig), and for *C*. *vishnui* it was 1165–1185 μs/cm(S7B Fig). Optimum Dissolved oxygen ranges from 2.67–3.80ppm (Fig 3A) in case of *An*. *stephensi*,for *A*. *albopictus* it was 4.6–7.4 ppm (S4A Fig), whereas 1–1.4ppm(S8A Fig) was the optimum range of DO for *C*.*vishnu*. COD value for *An*. *stephensi* ranges from 56–83 ppm (Fig 3B), while for *A*.*albopictus* it was 210–400 ppm (S4B Fig), for *C*.*vishnui*, it was 64-84ppm (S8B Fig). Optimum range of total alkalinity stands from 77-96ppm for *An*. *Stephensi*(Fig 4A), for *A*. *albopictus* it was 80–160 ppm (S5A Fig), for *C*. *vishnui* it was 430-453ppm (S9A Fig) and the optimum range of Hardness of the water for *An*. *stephensi* was 138-170ppm (Fig 4B), for *A*. *albopictus* it was 60-80ppm (S5B Fig) and for *C*. *vishnui* it was within the 268-283ppm (S9B Fig). *An*. *stephensi* habitats were found to be evenly distributed in between 0.448–0.78 ppm (Fig 5A) of Nitrate nitrogen content whereas for *A*. *albopictus* it was 1.02–1.456ppm.(S6A Fig), and for *C*. *vishnui* it was 0.98–1.047 ppm (S10A Fig). Ammonia nitrogen content ranges from 0.335–0.465 ppm (Fig 5B) for *An*. *stephensi*,on the other hand, it was 0.15–0.32ppm for *A*. *albopictus* (S6B Fig), and in case of *C*. *vishnui* it was 0.25–0.526ppm (S10B Fig).

Multiple logistic regression equations for three mosquito species are presented in Table 3. Higher larval density of *An*. *stephensi* is observed in lower pH, COD, T_alk_ along with higher Dissolved oxygen, Specific Conductance, Hardness, Ammonia nitrogen, Nitrate nitrogen(*p* = 1.37e-13, R^2^ = 0.9521). Higher pH, Specific Conductance, Dissolved oxygen, Ammonia nitrogen, Nitrate nitrogen and lower COD, T_alk_ and Hardness, are observed for higher density of *A*. *albopictus* (*p* = < 2.2e-16, R^2^ = 0.9737). On the other hand, higher larval density of *C*. *vishnui* is noted in higher pH, Dissolved oxygen, Specific Conductance, Hardness, Ammonia nitrogen, Nitrate nitrogen in combination with lower COD and lower T_alk_ (*p* = < 2.2e-16, R^2^ = 0.9862).All of these water quality variables are significant to elucidate the larval abundance.

**Table 3:** Multiple logistic regression equations for three mosquito larval densities related with significant water quality parameters.

Mosquito species	Multiple regression equation	P	R^2^
*An*. *stephensi*	Y = (-1.4114866)+(0.0531755*pH)+(0.0008908* SC)+(0.2025513*DO)+(0.0015446*COD)+(-0.0003189*T_alk_)+(-0.0060910*Hardness)+(-0.3545789*NN)+(3.5869414*AN)	1.37e-13	0.9521
*A*. *albopictus*	Y = (-2.8276547)+(0.3177430*pH)+(0.0004185* SC)+(-0.0398107*DO)+(-0.0011261*COD) +(0.0007373*T_alk_)+(0.0094951*Hardness)+(0.2109129*NN)+(0.7432535*AN)	< 2.2e-16	0.9737
*C*. *vishnui*	Y = (-3.697997)+(0.029618*pH)+(0.002498* SC.)+(0.004804*DO)+(0.002054*COD)+(-0.004239*T_alk_)+(0.005011*Hardness)+(1.173969*NN) +(-0.029614*AN)	< 2.2e-16	0.9862

Notes: Y = density of respective mosquito larvae.

### Inter-species association of mosquito larvae

**Fig 6:**
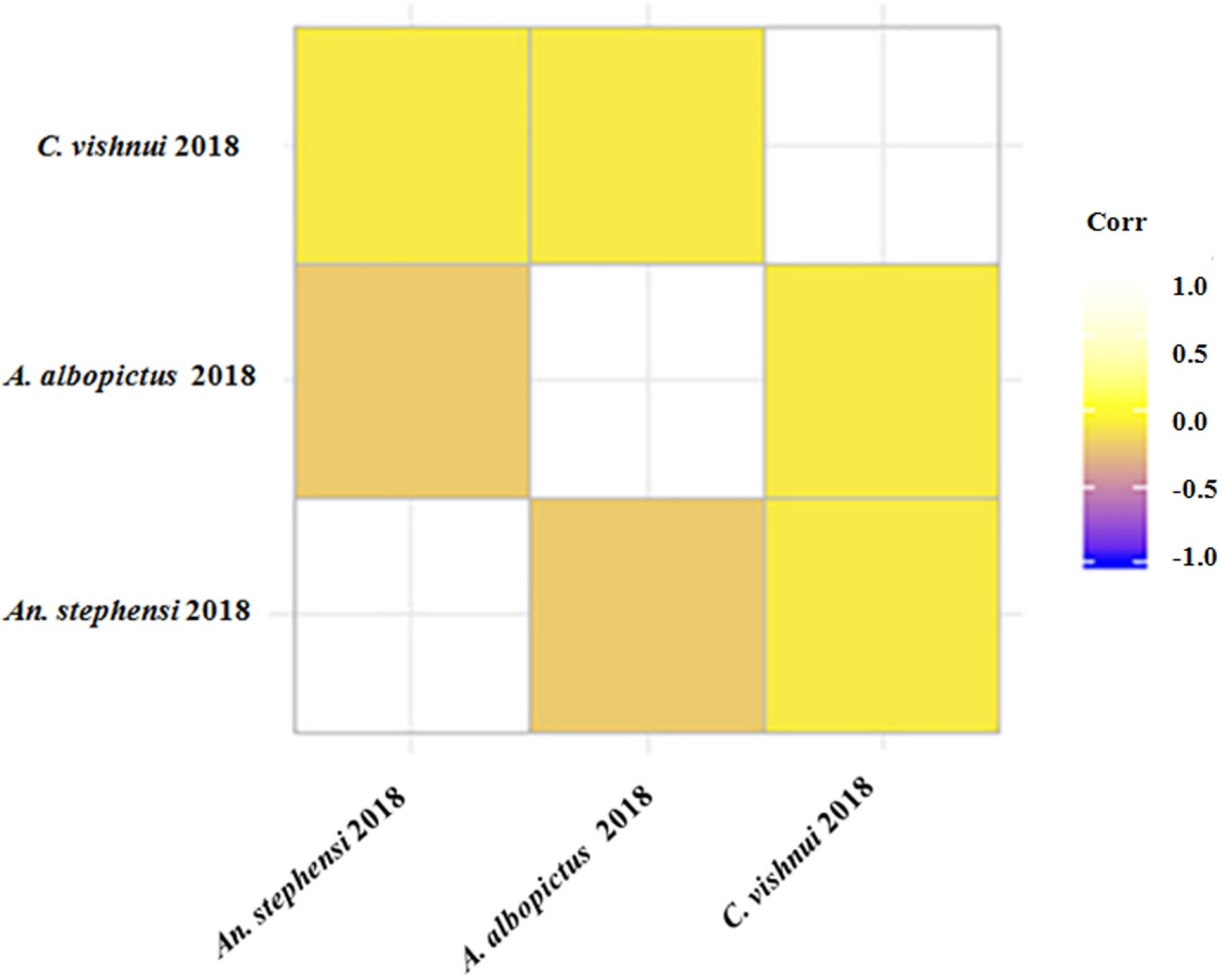
Spearmen correlation with ggcorrplot represents the negative correlation of all the three mosquito species with each other.

Spearman correlation revealed that occurrence of all the three mosquitoes (*An*. *stephensi*, *C*. *vishnui and A*. *albopictus*) was negatively correlated with each other due to the presence of different water quality parameters in different mosquito habitats. The results of analysis are given in Table 4. Correlations are plotted and presented in Fig 6.

**Table 4:** Inter species correlation among three mosquitoes.

Mosquito larvae	Rho value for inter correlation in 2018		
	*An*. *stephensi*	*A*. *albopictus*	*C*. *vishnui*
*An*. *stephensi*	1	-0.099	-0.194
*A*. *albopictus*	-0.099	1	-0.247
*C*. *vishnui*	-0.194	-0.247	1

### Correlation of water quality parameters and larval density with years for each larval habitat

Spearmen rank correlation exhibited a positive correlation of larval density and water quality parameters between two sampling years at each spot for three mosquito species (Table 5).

**Table 5:** Correlation of anopheline larval density and water quality parameters for each habitat of thirty selected habitats of two districts (KMC and Puruliya) within sampling years (2017 and 2018).

Districts	Areas	Habitats	Rho value of Spearmen Rank Correlation Test		
			Spot 1 2018	Spot 2 2018	Spot 3 2018
KMC	Borough I	Spot 1 2017	0.988		
		Spot 2 2017		1	
		Spot 3 2017			1
	Borough IV	Spot 1 2017	1		
		Spot 2 2017		1	
		Spot 3 2017			0.988
	Borough VII	Spot 1 2017	1		
		Spot 2 2017		0.988	
		Spot 3 2017			0.988
	Borough XI	Spot 1 2017	1		
		Spot 2 2017		0.988	
		Spot 3 2017			1
	Borough XV	Spot 1 2017	1		
		Spot 2 2017		0.988	
		Spot 3 2017			1
Puruliya	Puruliya I	Spot 1 2017	1		
		Spot 2 2017		0.988	
		Spot 3 2017			0.564
	Puruliya II	Spot 1 2017	1		
		Spot 2 2017		0.988	
		Spot 3 2017			1
	Arsha	Spot 1 2017	1		
		Spot 2 2017		0.988	
		Spot 3 2017			1
	Jhalda II	Spot 1 2017	0.988		
		Spot 2 2017		0.988	
		Spot 3 2017			0.988
	Balarampur	Spot 1 2017	1		
		Spot 2 2017		0.988	
		Spot 3 2017			1

Correlations of larval density of *C*.*vishnui* and *A*. *albopictus* and water quality parameters for each habitat within two sampling years (2017 and 2018) are presented in supplementary information file (S6 & S7 Tables).

## Discussion

Water quality parameters, which were analysed in our present study, have significant effects on density of medicinally important mosquitoes responsible for several mosquito borne diseases in West Bengal. Pearson correlation analysis exhibited that larval density of *An*. *stephensi* is negatively correlated with pH of the water but larval densities of *C*.*vishnui* and *A*. *albopictus* are positively correlated with pH. In the issue of larval density, *An*. *stephensi*, *A*. *albopictus* and *C*. *vishnui*, have positive correlation with specific conductance of the water. Dissolved oxygen is another significant parameter for larval density that has positive correlation with all three kinds of mosquitoes. Chemical Oxygen Demand is also a determining factor for larval occurrence which has negative correlation with all three mosquito species. Total alkalinity showed negative correlation with larval occurrence of all three types. It is widely known that hardness of water is a significant parameter for larval occurrence, and in our research, a positive correlation was noticed with larval density of *C*. *vishnui* and *An*. *stephensi* but a negative correlation was observed in the case of *A*.*albopictus*. Ammonia nitrogen and nitrate nitrogen content of water also plays a significant part for larval occurrence, and both have shown positive correlations with larval density of three types of mosquitoes. Bashar *et al*. [15] have reported the significance of water quality parameters in 2016 in Bangladesh. Our results of Pearson correlation test have validated their experimental data completely. A report about life stages of *Anopheles sp*. mosquito interpreted that a pH value that ranges from 6.8–7.2 is generally preferred by *Anopheles* mosquito for breeding [16]. 2D kernel test and Contour plot mapping of our study in two sampling years revealed that the optimum range of pH for *An*. *stephensi* was 6.31–6.90. It was reported that abundance of *An*. *stephensi* mosquito positively associated with Dissolved Oxygen [17, 18]. Sunish and Reuben [19] observed that Dissolved oxygen had a negative relationship with immature larval density in 2001. Amerasinghe *et al*. [20] determined that Dissolved oxygen is a major contributor to the mosquito larval habitat. In our study, Dissolved oxygen is also a major positive factor for density of mosquito larvae. Kengluecha *et al*. [21] reported in 2005 that Water Hardness is the good predictor for *Anopheles* in tropical climate where temperature is on the higher side and demonstrated a requirement of higher total hardness for the dominance of *A*.*minimus*. Similarly, our present research work clearly indicates that Water Hardness stands a good positive factor for larval density of *An*. *stephensi* and *C*. *vishnui* but this factor is not positive in case of larval density of *A*. *albopictus*. Contour plot analysis suggested that optimum range of Water Hardness for *An*. *stephensi* was138-170ppm and 268–283 ppm for *C*.*vishnui*. Comparatively lower range of Water hardness (60–80 ppm) requirement was found for *A*. *albopictus*. Bashar *et al*.[15] also reported lower requirement of Water Hardness for *Aedes* sp. larvae for its habitat selection which validated our data. Bradley and Kutz [22] reported positive co-relation of Ammonia nitrogen with larval abundance and the result of our present research corroborates with former workers. In a study, Sanford *et al*.[23], observed high Ammonia nitrogen requirement for *Culex* sp. larval abundance in wetland. Similarly, in our research, we have found that *C*. *vishnui* requires highest amount of Ammonia nitrogen, and in this case our observation matches with the observation of other researchers in the past. It was reported that municipal waste water effluence with high Ammonia nitrogen content significantly increases vegetation and mosquito population in wetland [24]. Increased organic pollution due to contamination from human wastes and agrochemicals is responsible for the change of physico-chemical property of the surface water. In our present study, we have detected a spot- wise positive correlation of larval density and water quality parameters within two sampling years for three species of mosquito. This study indicates that environmental pollution, which changes the water quality, can be a determining factor for mosquito population density. Inter- correlation study clearly demonstrates that all three (*An*. *stephensi*, *C*.*vishnui* and *A*. *albopictus*) disease transmitting mosquitoes are in negative correlation with each other due to the presence of different water quality parameters in different mosquito habitats.

After forgoing discussion, we may come to the conclusion that analysis in the laboratory of selected samples from different vulnerable localities in West Bengal helps us to have a good understanding about the parameters of the surface water that are suitable for oviposition of disease transmitting mosquitoes and to comprehend the larval density of respective mosquitoes. In the context of growing mosquito population as well as increased mosquito borne diseases, the findings of this research might be proved to be highly beneficial in the adoption of mosquito control program by environmental management, and accordingly, appropriate plans can be designed to control the larval population density. Moreover, water pollution is a positive indicator of larval density. Furthermore, our research establishes the fact that larval habitat of one disease transmitting mosquito is not suitable for other two. It needs to be emphasized that this kind of work has not been carried out much, this research work is relatively new in the mosquito borne disease affected areas of West Bengal where correlations between water quality parameters with larval densities of primary vectors of malaria, dengue and encephalitis were measured by the advance version of R (3.4.4). This technique can be useful in other zones of the world for larval population and density analysis, which is a pre- requisite criterion for management of mosquito borne human diseases.

## Supporting information

S1 Fig Map of the studied districts (marked with color) in West Bengal, India (Map was drawn and edited with MS paint, following image obtained from the open source: http://landsatlook.usgs.gov/viewer.html. (TIF)

S2 Fig Sample photographs of mosquito larval habitats A. *An*. *stephensi* B. *A*. *albopictus* and C. *C*. *vishnui*. (TIF)

S3 Fig **Contour plot graph exhibiting correlation of (A) pH and (B) Specific Conductance with larval density of *A*. *albopictus***.
(TIF)

S4 Fig Contour plot graph exhibiting correlation of (A) DO and (B) COD with larval density of *A*. *albopictus*.
(TIF)

S5 Fig Contour plot graph exhibiting correlation of (A) Total alkalinity and (B) Hardness with larval density *A*. *albopictus*.
(TIF)

S6 Fig Contour plot graph exhibiting correlation of (A) Nitrate nitrogen and (B) Ammonia nitrogen with larval density of *A*. *albopictus*.
(TIF)

S7 Fig Contour plot graph exhibiting correlation of (A) pH and (B) Specific Conductance with larval density of *C*. *vishnui*.
(TIF)

S8 Fig Contour plot graph exhibiting correlation of (A) DO and (B) COD with larval density of *C*. *vishnui*.
(TIF)

S9 Fig Contour plot graph exhibiting correlation of (A) Total alkalinity and (B) Hardness with larval density *C*. *vishnui*.
(TIF)

S10 Fig Contour plot graph exhibiting correlation of (A) Nitrate nitrogen and (B) Ammonia nitrogen with larval density of *C*. *vishnui*.
(TIF)

S1 Table Total estimated larval population of three mosquito species in selected districts in two sampling years (2017 and 2018). (DOCX)

S2 Table Larval density index values of three different mosquito larvae species. (DOCX)

S3 Table Density of *C*. *vishnui* larvae in each habitat of selected ten blocks in two districts (Alipurduar and Burdwan) of West Bengal. (DOCX)

S4 Table Density of *A*. *albopictus* larvae in each habitat of selected ten areas in two districts (North 24 Parganas and Haora) of West Bengal. (DOCX)

S5 Table. Density of An *stephensi* larvae in each habitat of selected ten areas in two districts (KMC and Puruliya) of West Bengal. (DOCX)

S6 Table Correlation of *A*. *albopictus* larvae density and water quality parameters for each habitat of thirty selected habitats of two districts (N 24 Parganas and Haora) within sampling years (2017 and 2018). (DOCX)

S7 Table Correlation of *C*. *vishnui* larvae density and water quality parameters for each habitat of thirty selected habitats of two districts (Alipurduar and Burdwan) within sampling years (2017 and 2018). (DOCX)
