# Supplementary figures and images for: In target areas where human mosquito-borne diseases are diagnosed, the inclusion of the pre-adult mosquito aquatic niches parameters will improve the integrated mosquito control program
Source: PLoS Negl Trop Dis. 2020 Aug 14;14(8):e0008605. doi: 10.1371/journal.pntd.0008605 (PMC7449462; doi:10.1371/journal.pntd.0008605)

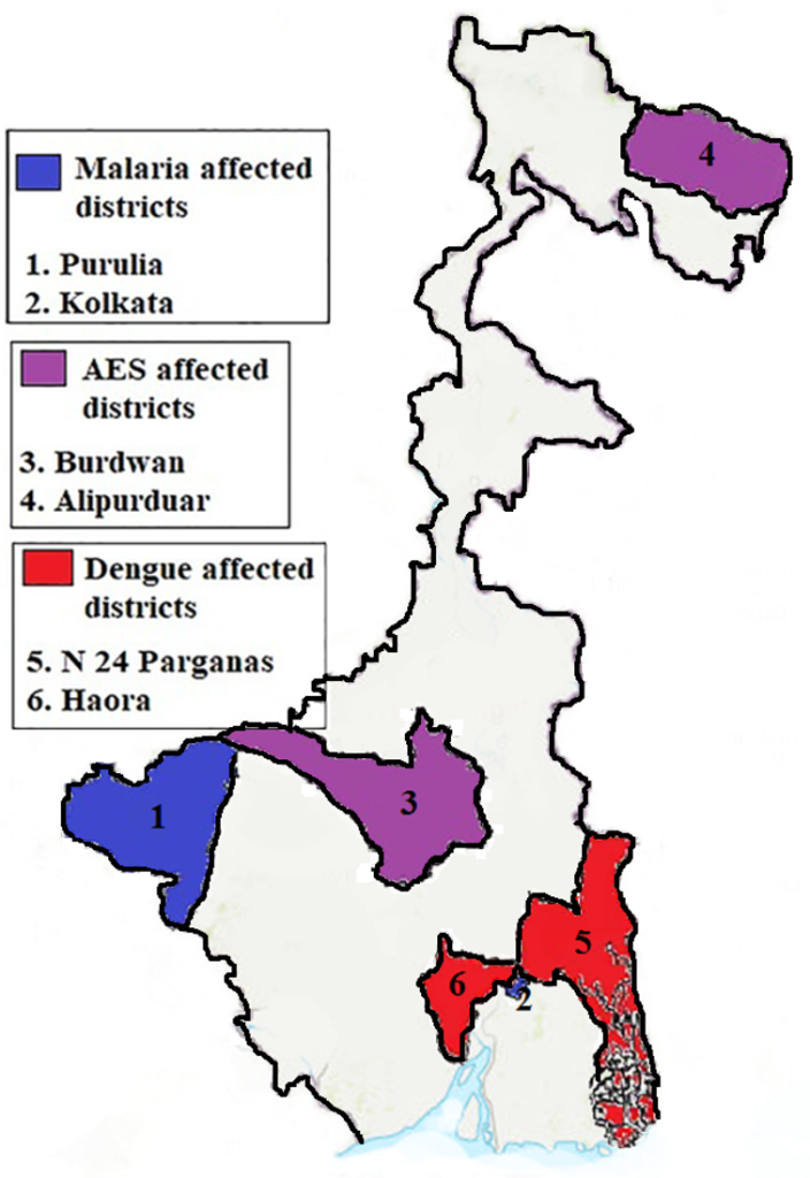

Supplement: S1 Fig — (TIF) [file pntd.0008605.s001.tif]

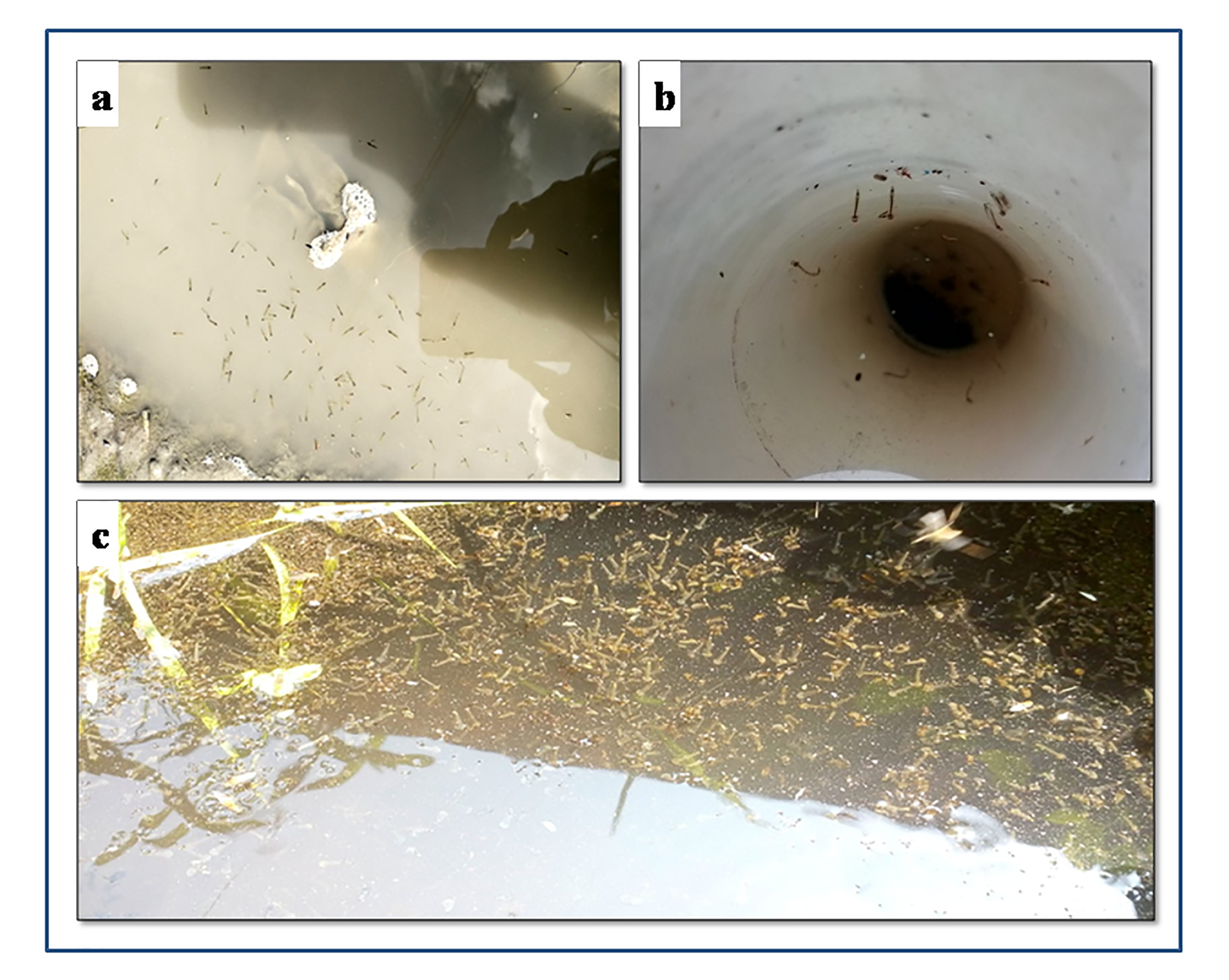

Supplement: S2 Fig — (TIF) [file pntd.0008605.s002.tif]

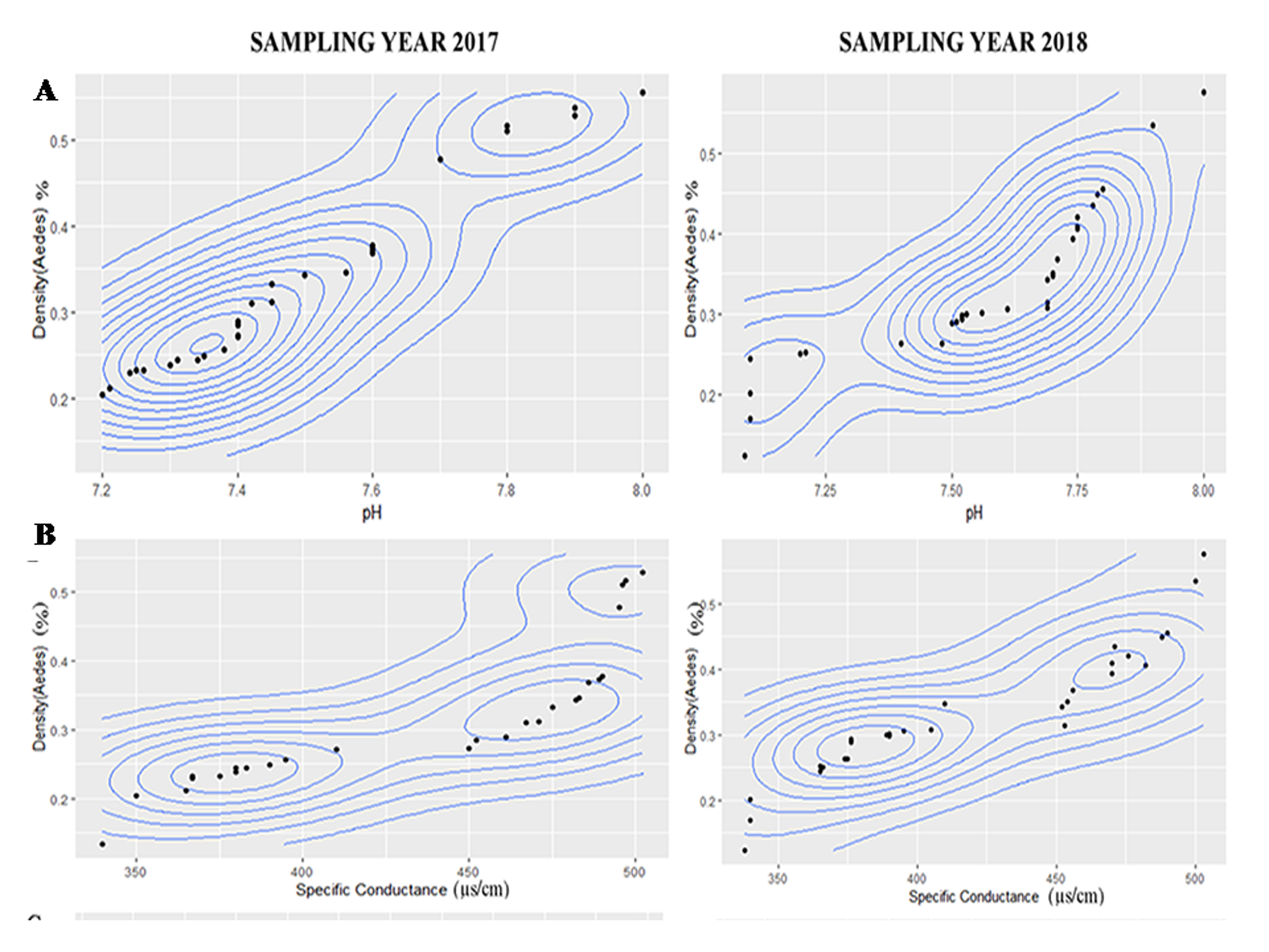

Supplement: S3 Fig — Contour plot graph exhibiting correlation of (A) pH and (B) Specific Conductance with larval density of A. albopictus. (TIF) [file pntd.0008605.s003.tif]

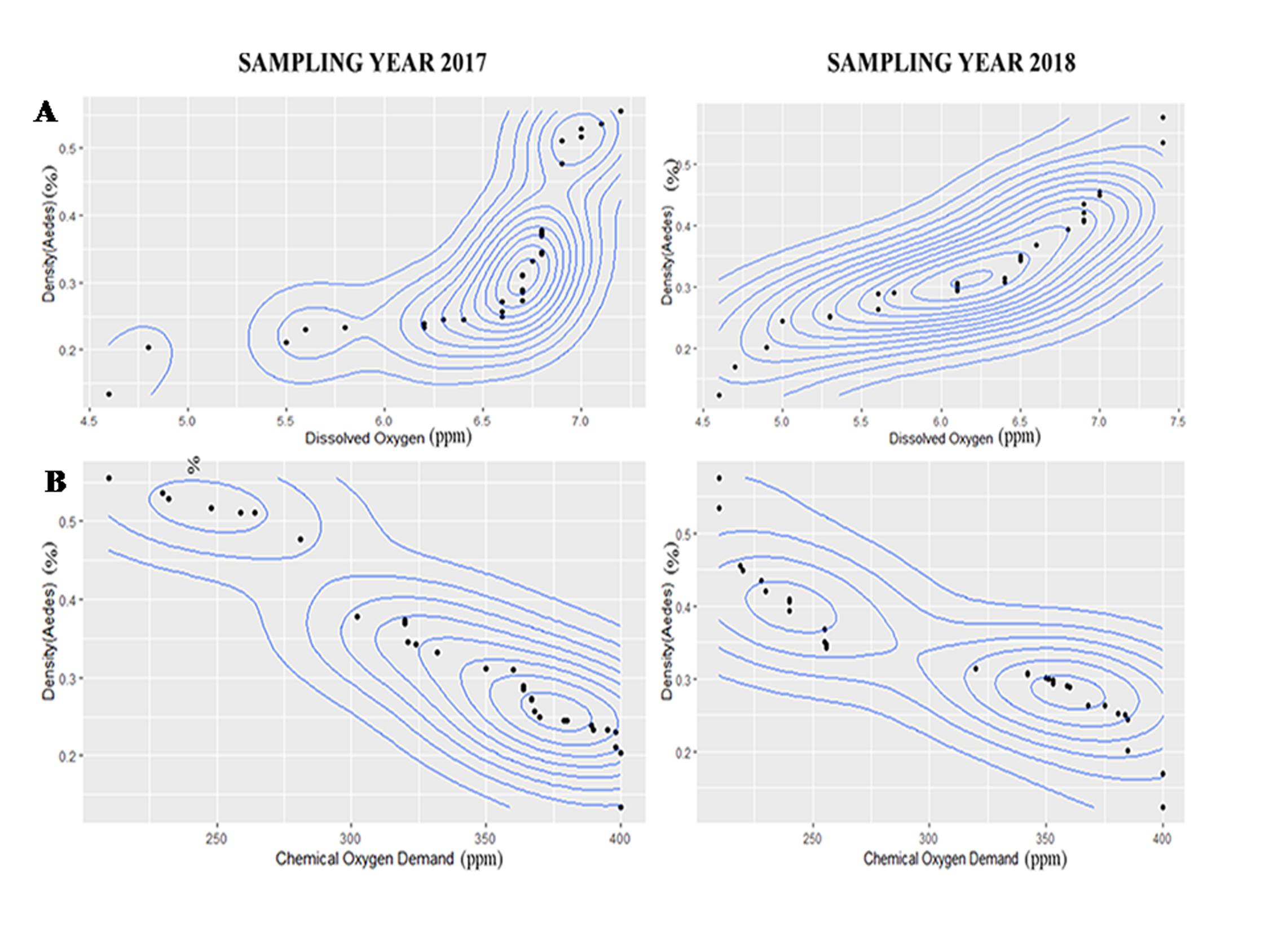

Supplement: S4 Fig — Contour plot graph exhibiting correlation of (A) DO and (B) COD with larval density of A. albopictus. (TIF) [file pntd.0008605.s004.tif]

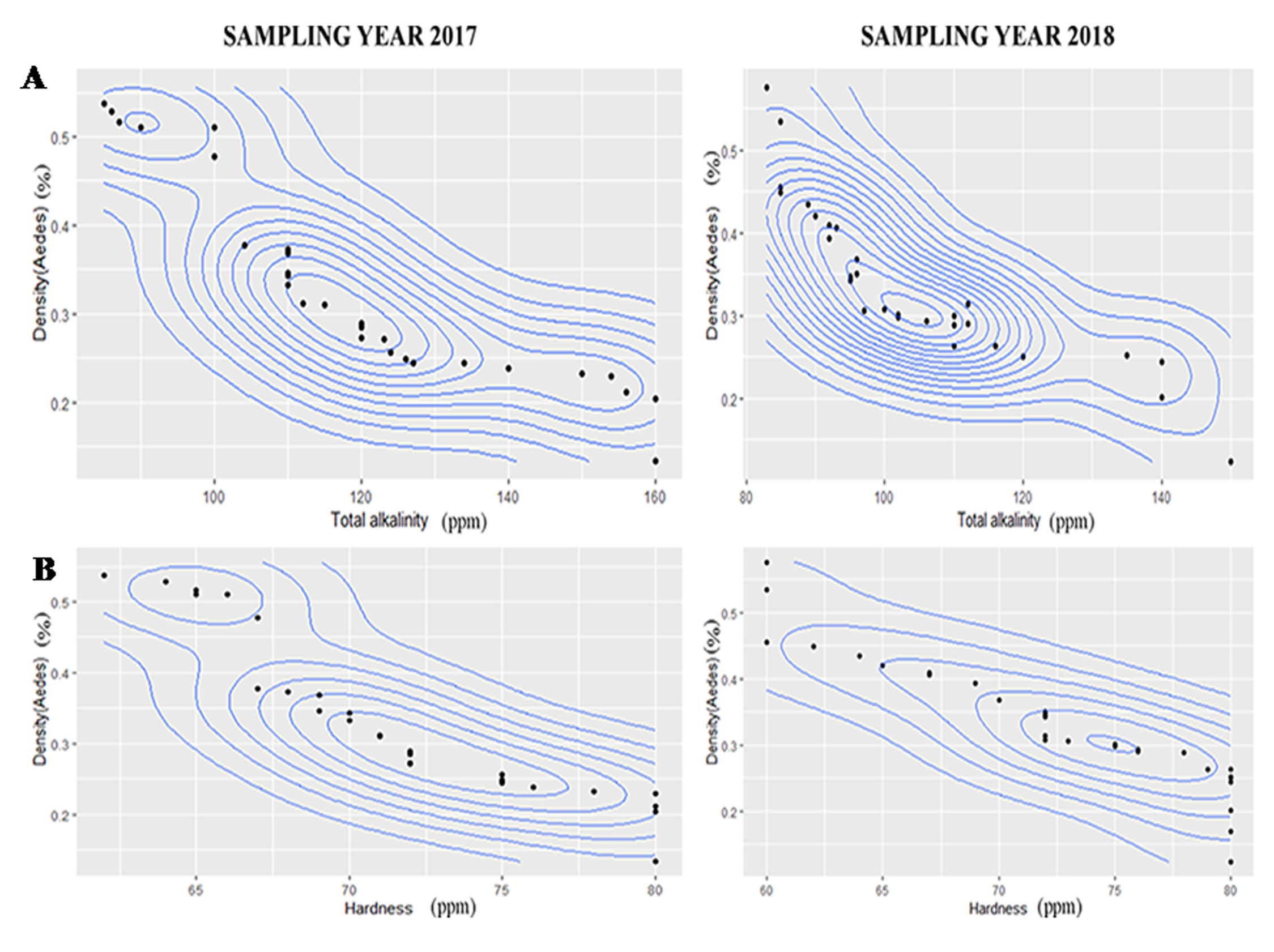

Supplement: S5 Fig — Contour plot graph exhibiting correlation of (A) Total alkalinity and (B) Hardness with larval density A. albopictus. (TIF) [file pntd.0008605.s005.tif]

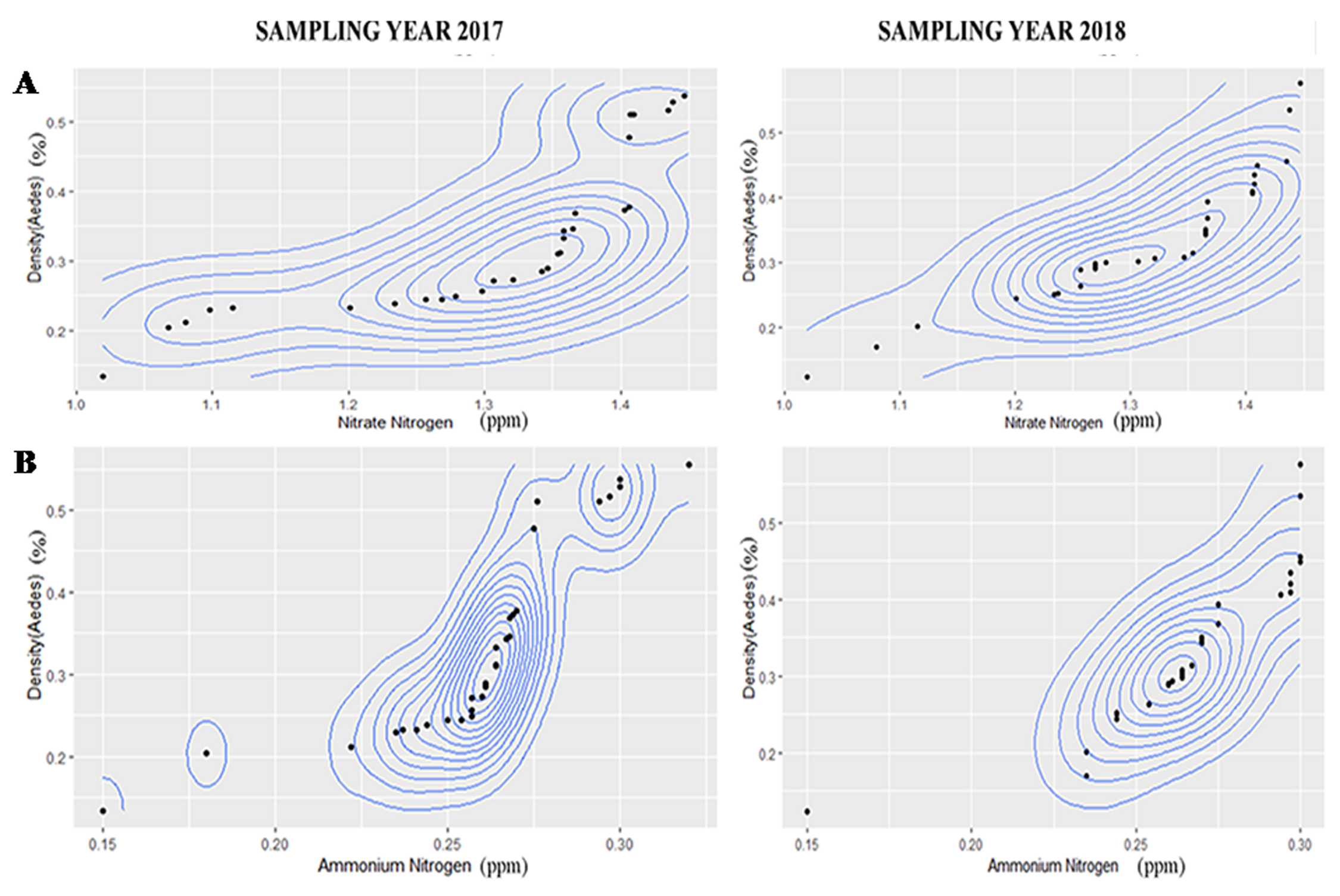

Supplement: S6 Fig — Contour plot graph exhibiting correlation of (A) Nitrate nitrogen and (B) Ammonia nitrogen with larval density of A. albopictus. (TIF) [file pntd.0008605.s006.tif]

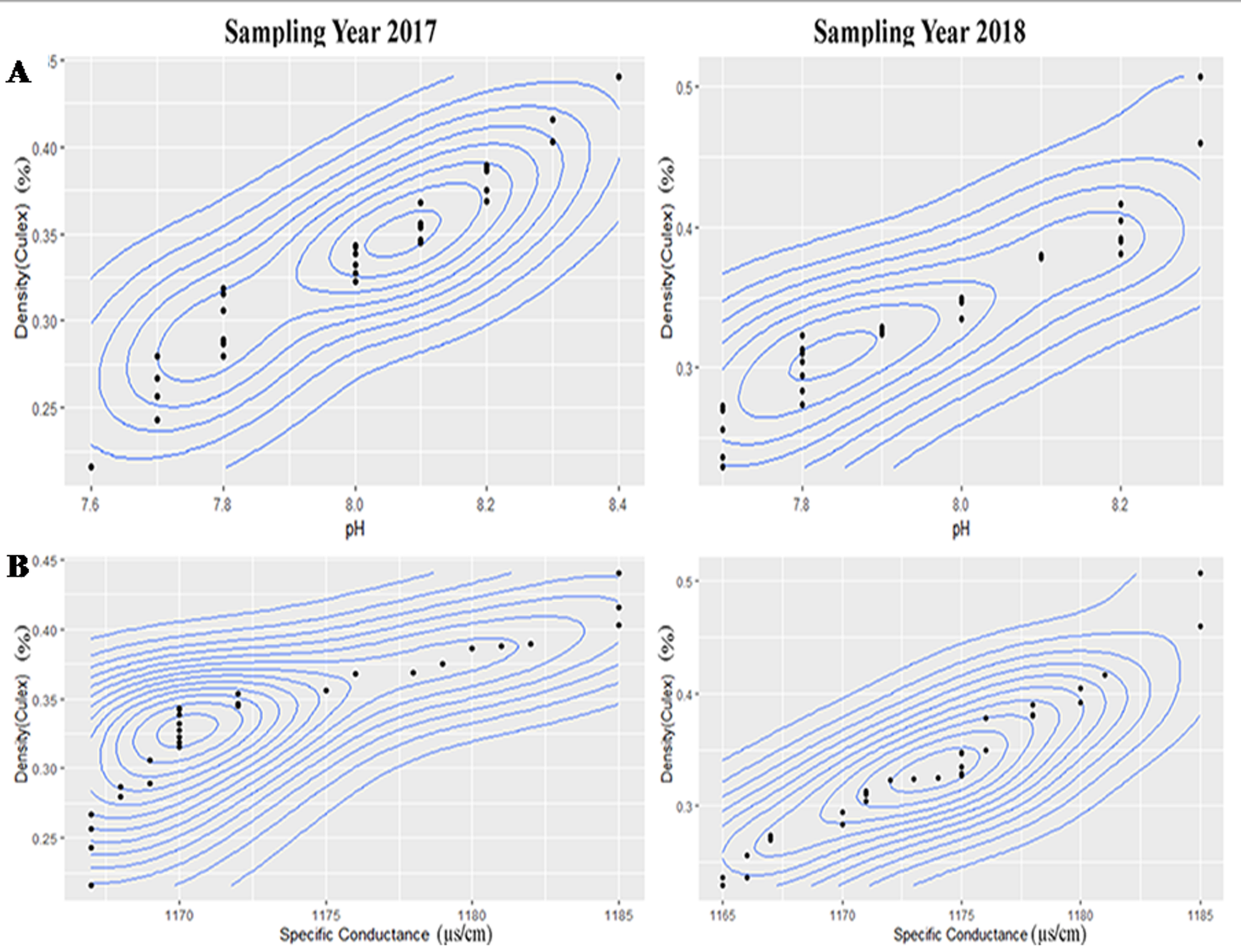

Supplement: S7 Fig — Contour plot graph exhibiting correlation of (A) pH and (B) Specific Conductance with larval density of C. vishnui. (TIF) [file pntd.0008605.s007.tif]

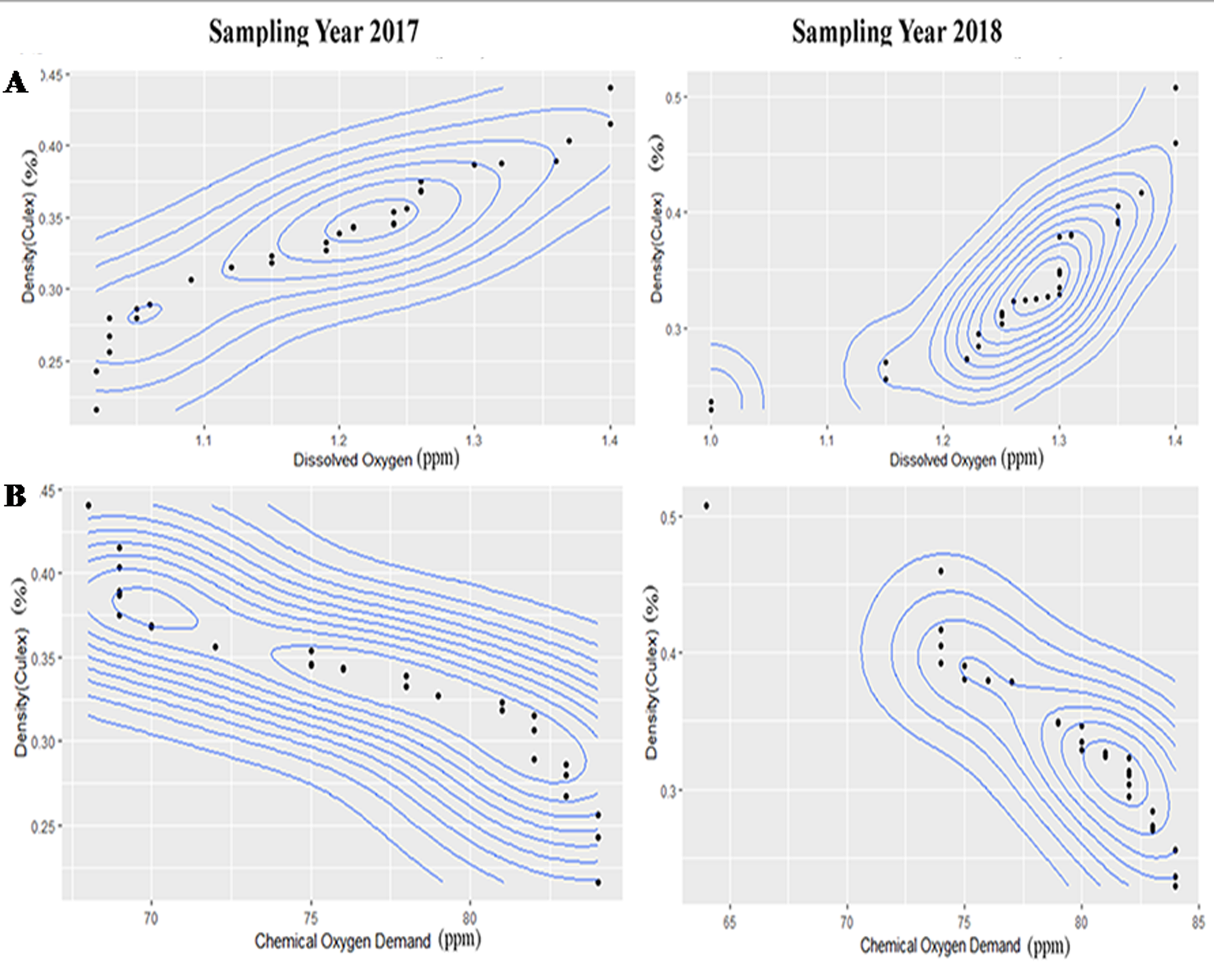

Supplement: S8 Fig — Contour plot graph exhibiting correlation of (A) DO and (B) COD with larval density of C. vishnui. (TIF) [file pntd.0008605.s008.tif]

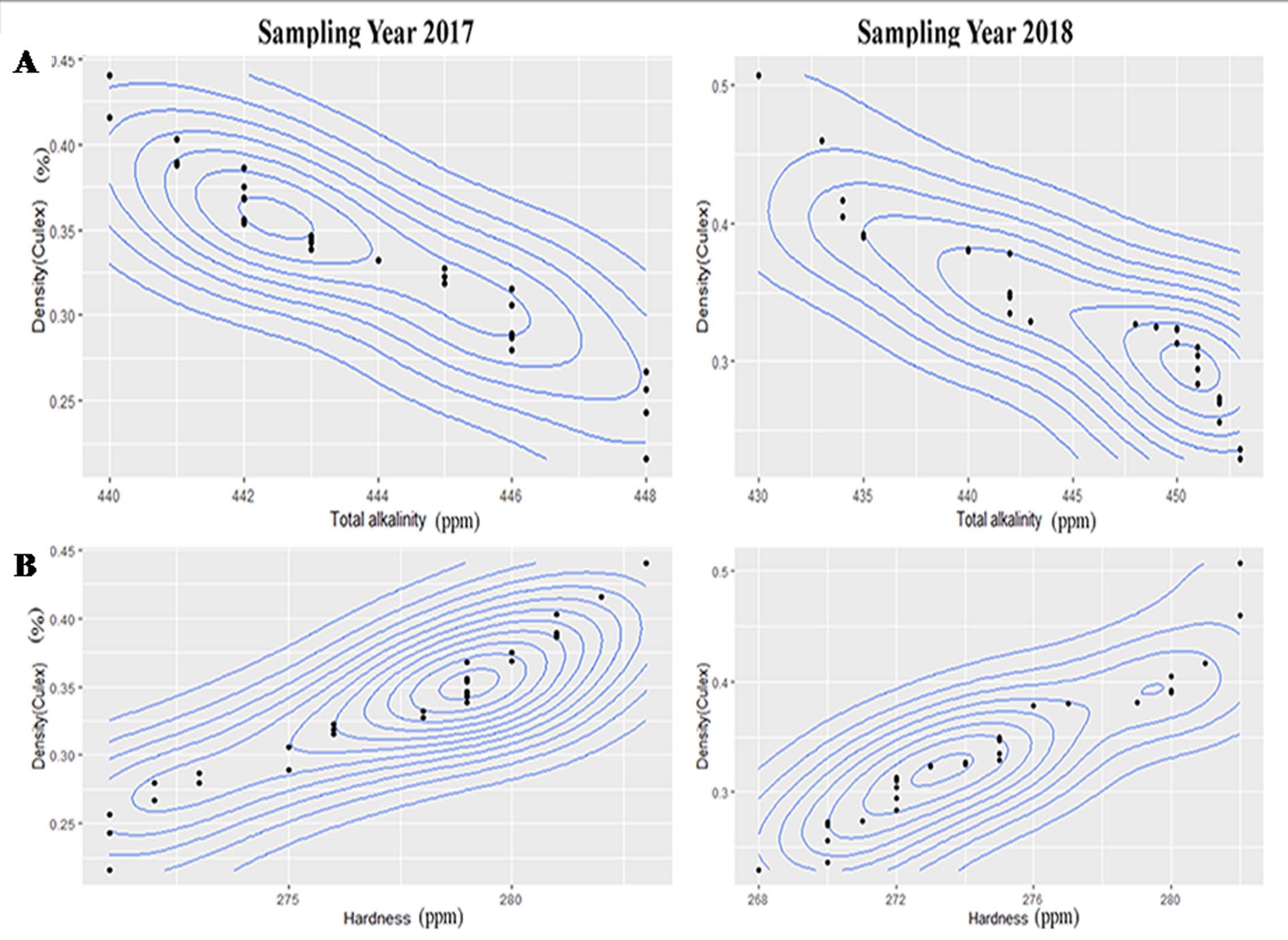

Supplement: S9 Fig — Contour plot graph exhibiting correlation of (A) Total alkalinity and (B) Hardness with larval density C. vishnui. (TIF) [file pntd.0008605.s009.tif]

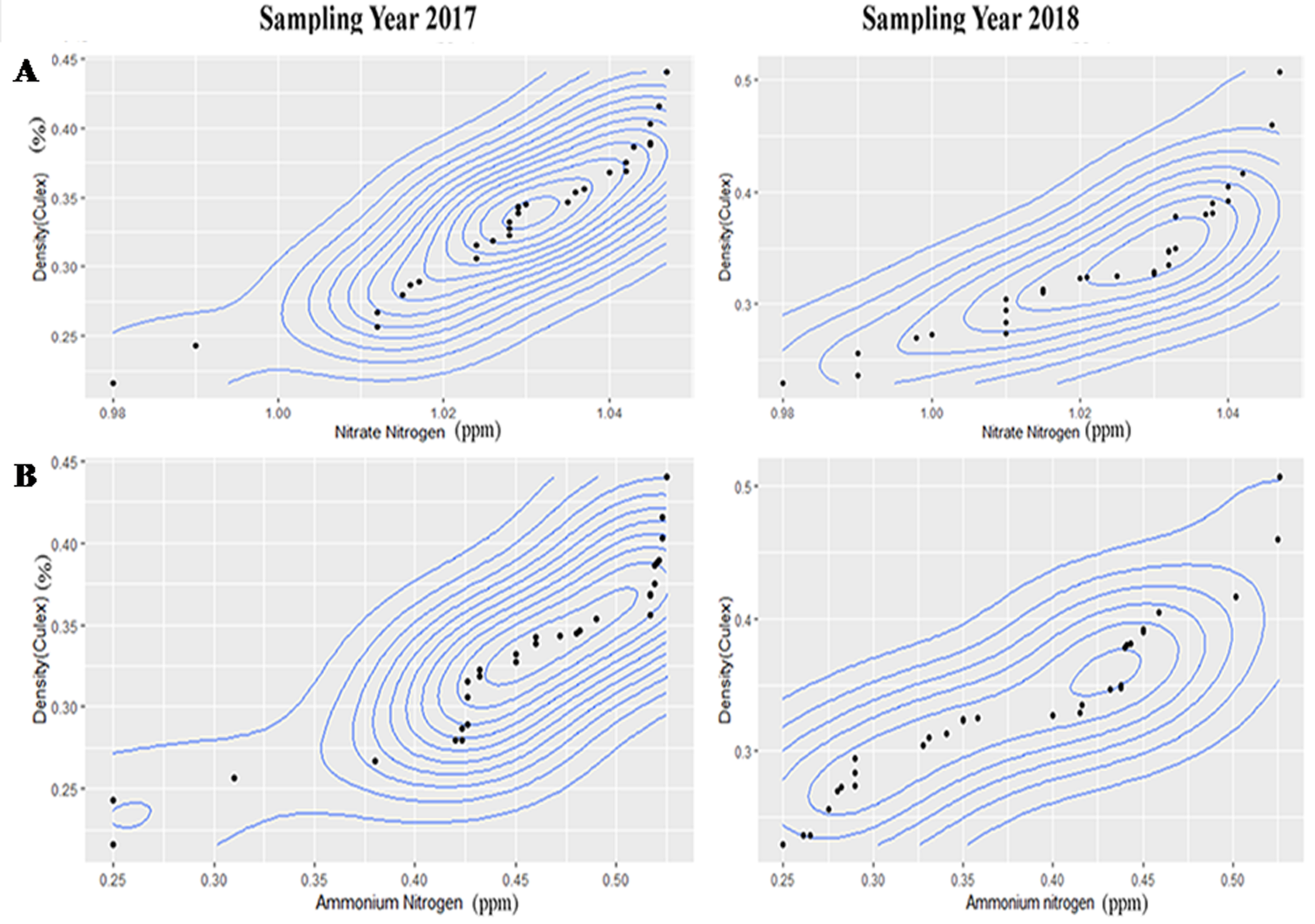

Supplement: S10 Fig — Contour plot graph exhibiting correlation of (A) Nitrate nitrogen and (B) Ammonia nitrogen with larval density of C. vishnui. (TIF) [file pntd.0008605.s010.tif]
