# Supplementary material for: In target areas where human mosquito-borne diseases are diagnosed, the inclusion of the pre-adult mosquito aquatic niches parameters will improve the integrated mosquito control program
Source: PLoS Negl Trop Dis. 2020 Aug 14;14(8):e0008605. doi: 10.1371/journal.pntd.0008605 (PMC7449462; doi:10.1371/journal.pntd.0008605)
Supplement: S1 Table — (DOCX) [file pntd.0008605.s011.docx]

Table S1 Total estimated larval population of three mosquito species in selected districts in two sampling years (2017 and 2018)

| Mosquito larvae species | Estimated total larvae population of selected areas of respective districts of West Bengal in two sampling years | | | |
| --- | --- | --- | --- | --- |
|  | **2017** | | **2018** | |
|  | **Districts/ Areas** | | **Districts/ Areas** | |
| *An. stephensi* | **KMC Area** | **Puruliya** | **KMC Area** | **Puruliya** |
|  | 2339 | 2692.25 | 2439.32 | 2853.4 |
| *C. vishnui* | **Alipurduar** | **Burdwan** | **Alipurduar** | **Burdwan** |
|  | 24085.17 | 21738.5 | 25834.1 | 22549.67 |
| *A. albopictus* | **North 24 Parganas** | **Haora** | **North 24Parganas** | **Haora** |
|  | 470.216 | 873.60 | 604.983 | 850.56 |
