# Supplementary material for: In target areas where human mosquito-borne diseases are diagnosed, the inclusion of the pre-adult mosquito aquatic niches parameters will improve the integrated mosquito control program
Source: PLoS Negl Trop Dis. 2020 Aug 14;14(8):e0008605. doi: 10.1371/journal.pntd.0008605 (PMC7449462; doi:10.1371/journal.pntd.0008605)
Supplement: S2 Table — (DOCX) [file pntd.0008605.s012.docx]

Table S2 Larval density index values of three different mosquito larvae species.

| **Mosquito larvae**  **species** | **Larval Density Index (LDI) value of three mosquito species in two sampling years** | |
| --- | --- | --- |
|  | **2017** | **2018** |
| *C. vishnui* | 87.78 | 87.76 |
| *A. albopictus* | 2.57 | 2.64 |
| *An. stephensi* | 9.63 | 9.59 |
