# Supplementary material for: In target areas where human mosquito-borne diseases are diagnosed, the inclusion of the pre-adult mosquito aquatic niches parameters will improve the integrated mosquito control program
Source: PLoS Negl Trop Dis. 2020 Aug 14;14(8):e0008605. doi: 10.1371/journal.pntd.0008605 (PMC7449462; doi:10.1371/journal.pntd.0008605)
Supplement: S3 Table — (DOCX) [file pntd.0008605.s013.docx]

Table S3 Density of *C. vishnui* larvae in each habitat of selected ten blocks in two districts (Alipurduar and Burdwan) of West Bengal.

| Districts | Selected areas | Selected larval habitats | Density/habitat in sampling years | |
| --- | --- | --- | --- | --- |
|  |  |  | **2017** | **2018** |
| Alipurduar | Alipurduar I | Spot 1 | 0.216 | 0.229 |
|  |  | Spot 2 | 0.368 | 0.379 |
|  |  | Spot 3 | 0.415 | 0.390 |
|  | Alipurduar II | Spot 1 | 0.403 | 0.273 |
|  |  | Spot 2 | 0.353 | 0.347 |
|  |  | Spot 3 | 0.242 | 0.378 |
|  | Falakata | Spot 1 | 0.386 | 0.323 |
|  |  | Spot 2 | 0.327 | 0.326 |
|  |  | Spot 3 | 0.286 | 0.349 |
|  | Kumargram | Spot 1 | 0.279 | 0.324 |
|  |  | spot 2 | 0.279 | 0.329 |
|  |  | spot 3 | 0.440 | 0.346 |
|  | Kalchini | spot 1 | 0.389 | 0.272 |
|  |  | spot 2 | 0.343 | 0.310 |
|  |  | spot 3 | 0.267 | 0.416 |
| Burdwan | Burdwan I | spot 1 | 0.315 | 0.283 |
|  |  | spot 2 | 0.338 | 0.335 |
|  |  | spot 3 | 0.346 | 0.380 |
|  | Galsi II | spot 1 | 0.306 | 0.236 |
|  |  | spot 2 | 0.318 | 0.255 |
|  |  | spot 3 | 0.375 | 0.507 |
|  | Rayna II | spot 1 | 0.256 | 0.236 |
|  |  | spot 2 | 0.356 | 0.304 |
|  |  | spot 3 | 0.387 | 0.459 |
|  | Kalna II | spot 1 | 0.344 | 0.270 |
|  |  | spot 2 | 0.332 | 0.324 |
|  |  | spot 3 | 0.322 | 0.405 |
|  | Katwa I | spot 1 | 0.289 | 0.294 |
|  |  | spot 2 | 0.368 | 0.313 |
|  |  | spot 3 | 0.342 | 0.392 |
