# Supplementary material for: In target areas where human mosquito-borne diseases are diagnosed, the inclusion of the pre-adult mosquito aquatic niches parameters will improve the integrated mosquito control program
Source: PLoS Negl Trop Dis. 2020 Aug 14;14(8):e0008605. doi: 10.1371/journal.pntd.0008605 (PMC7449462; doi:10.1371/journal.pntd.0008605)
Supplement: S4 Table — (DOCX) [file pntd.0008605.s014.docx]

Table S4 Density of *A. albopictus* larvae in each habitat of selected ten areas in two districts (North 24 Parganas and Haora) of West Bengal.

| Districts | Selected areas | Selected larval habitats | Density/ habitat in sampling years | |
| --- | --- | --- | --- | --- |
| North 24 Parganas |  |  |  |  |
|  |  |  | **2017** | **2018** |
|  | Basirhat | Spot 1 | 0.278 | 0.313 |
|  |  | Spot 2 | 0.348 | 0.251 |
|  |  | Spot 3 | 0.372 | 0.435 |
|  | Haroa | Spot 1 | 0.276 | 0.408 |
|  |  | Spot 2 | 0.318 | 0.244 |
|  |  | Spot 3 | 0.404 | 0.347 |
|  | Bangaon | Spot 1 | 0.142 | 0.575 |
|  |  | Spot 2 | 0.357 | 0.123 |
|  |  | Spot 3 | 0.500 | 0.300 |
|  | Habra | Spot 1 | 0.211 | 0.454 |
|  |  | spot 2 | 0.381 | 0.293 |
|  |  | spot 3 | 0.406 | 0.252 |
|  | Swarupnagar | spot 1 | 0.241 | 0.367 |
|  |  | spot 2 | 0.241 | 0.343 |
|  |  | spot 3 | 0.516 | 0.289 |
| Haora | Bally | spot 1 | 0.238 | 0.305 |
|  |  | spot 2 | 0.263 | 0.406 |
|  |  | spot 3 | 0.497 | 0.288 |
|  | Sankrail | spot 1 | 0.231 | 0.448 |
|  |  | spot 2 | 0.347 | 0.349 |
|  |  | spot 3 | 0.420 | 0.201 |
|  | Uluberia | spot 1 | 0.226 | 0.297 |
|  |  | spot 2 | 0.241 | 0.168 |
|  |  | spot 3 | 0.531 | 0.534 |
|  | Domjur | spot 1 | 0.219 | 0.419 |
|  |  | spot 2 | 0.287 | 0.262 |
|  |  | spot 3 | 0.493 | 0.262 |
|  | Bagnan I | spot 1 | 0.232 | 0.299 |
|  |  | spot 2 | 0.284 | 0.307 |
|  |  | spot 3 | 0.483 | 0.393 |
