# Supplementary material for: In target areas where human mosquito-borne diseases are diagnosed, the inclusion of the pre-adult mosquito aquatic niches parameters will improve the integrated mosquito control program
Source: PLoS Negl Trop Dis. 2020 Aug 14;14(8):e0008605. doi: 10.1371/journal.pntd.0008605 (PMC7449462; doi:10.1371/journal.pntd.0008605)
Supplement: S5 Table. Density of An — (DOCX) [file pntd.0008605.s015.docx]

Table S5 Density of *An. stephensi* larvae in each habitat of selected ten areas in two districts (KMC and Puruliya) of West Bengal.

| **Districts** | **Selected areas** | Selected habitats | Density/habitat in sampling years | |
| --- | --- | --- | --- | --- |
|  |  |  | **2017** | **2018** |
| KMC | Borough I | Spot 1 | 0.278 | 0.459 |
|  |  | Spot 2 | 0.348 | 0.246 |
|  |  | Spot 3 | 0.372 | 0.294 |
|  | Borough IV | Spot 1 | 0.276 | 0.285 |
|  |  | Spot 2 | 0.318 | 0.224 |
|  |  | Spot 3 | 0.404 | 0.489 |
|  | Borough VII | Spot 1 | 0.142 | 0.275 |
|  |  | Spot 2 | 0.357 | 0.450 |
|  |  | Spot 3 | 0.500 | 0.275 |
|  | Borough XI | Spot 1 | 0.211 | 0.335 |
|  |  | Spot 2 | 0.381 | 0.453 |
|  |  | Spot 3 | 0.406 | 0.210 |
|  | Borough XV | Spot 1 | 0.241 | 0.363 |
|  |  | Spot 2 | 0.516 | 0.417 |
|  |  | Spot 3 | 0.238 | 0.218 |
| PURULIYA | Puruliya I | Spot 1 | 0.263 | 0.349 |
|  |  | Spot 2 | 0.497 | 0.281 |
|  |  | Spot 3 | 0.231 | 0.523 |
|  | Puruliya II | Spot 1 | 0.347 | 0.146 |
|  |  | Spot 2 | 0.420 | 0.330 |
|  |  | Spot 3 | 0.226 | 0.383 |
|  | Arsha | Spot 1 | 0.241 | 0.329 |
|  |  | Spot 2 | 0.531 | 0.286 |
|  |  | Spot 3 | 0.219 | 0.223 |
|  | Jhalda II | Spot 1 | 0.287 | 0.478 |
|  |  | Spot 2 | 0.493 | 0.298 |
|  |  | Spot 3 | 0.232 | 0.349 |
|  | Balarampur | Spot 1 | 0.284 | 0.393 |
|  |  | Spot 2 | 0.483 | 0.257 |
|  |  | Spot 3 | 0.241 | 0.281 |
