# Supplementary material for: In target areas where human mosquito-borne diseases are diagnosed, the inclusion of the pre-adult mosquito aquatic niches parameters will improve the integrated mosquito control program
Source: PLoS Negl Trop Dis. 2020 Aug 14;14(8):e0008605. doi: 10.1371/journal.pntd.0008605 (PMC7449462; doi:10.1371/journal.pntd.0008605)
Supplement: S6 Table — (DOCX) [file pntd.0008605.s016.docx]

Table S6 Correlation of *A. albopictus* larvae density and water quality parameters for each habitat of thirty selected habitats of two districts (N 24 Parganas and Haora) within sampling years (2017 and 2018).

| Districts | Areas | Habitats | Rho value of Spearmen Rank Correlation Test | | | |
| --- | --- | --- | --- | --- | --- | --- |
|  |  |  | Spot 1 2018 | Spot 2 2018 | | Spot 3 2018 |
| N24 PGS | Basirhat I | Spot 1 2017 | 0.903 |  | |  |
|  |  | Spot 2 2017 |  | 0.915 | |  |
|  |  | Spot 3 2017 |  |  | | 0.939 |
|  | Haroa | Spot 1 2017 | 0.924 |  | |  |
|  |  | Spot 2 2017 |  | 0.915 | |  |
|  |  | Spot 3 2017 |  |  | | 0.903 |
|  | Bangaon | Spot 1 2017 | 0.939 |  | |  |
|  |  | Spot 2 2017 |  | 0.915 | |  |
|  |  | Spot 3 2017 |  |  | | 0.951 |
|  | Habra | Spot 1 2017 | 0.939 | |  |  |
|  |  | Spot 2 2017 |  | | 0.939 |  |
|  |  | Spot 3 2017 |  | |  | 0.939 |
|  | Swarupnaga-r | Spot 1 2017 | 0.939 | |  |  |
|  |  | Spot 2 2017 |  | | 0.951 |  |
|  |  | Spot 3 2017 |  | |  | 0.939 |
| Haora | Bally | Spot 1 2017 | 0.939 | |  |  |
|  |  | Spot 2 2017 |  | | 1 |  |
|  |  | Spot 3 2017 |  | |  | 0.951 |
|  | Sankarail | Spot 1 2017 | 0.939 | |  |  |
|  |  | Spot 2 2017 |  | | 0.951 |  |
|  |  | Spot 3 2017 |  | |  | 0.951 |
|  | Uluberia | Spot 1 2017 | 0.939 | |  |  |
|  |  | Spot 2 2017 |  | | 0.903 |  |
|  |  | Spot 3 2017 |  | |  | 0.939 |
|  | Domjur | Spot 1 2017 | 0.939 | |  |  |
|  |  | Spot 2 2017 |  | | 0.951 |  |
|  |  | Spot 3 2017 |  | |  | 0.939 |
|  | Bagnan I | Spot 1 2017 | 0.939 | |  |  |
|  |  | Spot 2 2017 |  | | 0.951 |  |
|  |  | Spot 3 2017 |  | |  | 0.939 |
