# Supplementary material for: In target areas where human mosquito-borne diseases are diagnosed, the inclusion of the pre-adult mosquito aquatic niches parameters will improve the integrated mosquito control program
Source: PLoS Negl Trop Dis. 2020 Aug 14;14(8):e0008605. doi: 10.1371/journal.pntd.0008605 (PMC7449462; doi:10.1371/journal.pntd.0008605)
Supplement: S7 Table — (DOCX) [file pntd.0008605.s017.docx]

Table S7 Correlation of *C. vishnui* larvae density and water quality parameters for each habitat of thirty selected habitats of two districts (Alipurduar and Burdwan) within sampling years (2017 and 2018).

| Districts | Areas | Habitats | Rho value of Spearmen Rank Correlation Test | | | |
| --- | --- | --- | --- | --- | --- | --- |
|  |  |  | Spot 1 2018 | Spot 2 2018 | | Spot 3 2018 |
| Alipurduar | Alipurduar I | Spot 1 2017 | 1 |  | |  |
|  |  | Spot 2 2017 |  | 1 | |  |
|  |  | Spot 3 2017 |  |  | | 1 |
|  | Alipurduar II | Spot 1 2017 | 1 |  | |  |
|  |  | Spot 2 2017 |  | 1 | |  |
|  |  | Spot 3 2017 |  |  | | 1 |
|  | Falakata | Spot 1 2017 | 1 |  | |  |
|  |  | Spot 2 2017 |  | 1 | |  |
|  |  | Spot 3 2017 |  |  | | 1 |
|  | Kumargram | Spot 1 2017 | 1 | |  |  |
|  |  | Spot 2 2017 |  | | 1 |  |
|  |  | Spot 3 2017 |  | |  | 1 |
|  | Kalchini | Spot 1 2017 | 1 | |  |  |
|  |  | Spot 2 2017 |  | | 1 |  |
|  |  | Spot 3 2017 |  | |  | 1 |
| Burdwan | Burdwan I | Spot 1 2017 | 1 | |  |  |
|  |  | Spot 2 2017 |  | | 1 |  |
|  |  | Spot 3 2017 |  | |  | 1 |
|  | KalnaII | Spot 1 2017 | 1 | |  |  |
|  |  | Spot 2 2017 |  | | 1 |  |
|  |  | Spot 3 2017 |  | |  | 0.987 |
|  | Galsi II | Spot 1 2017 | 1 | |  |  |
|  |  | Spot 2 2017 |  | | 1 |  |
|  |  | Spot 3 2017 |  | |  | 1 |
|  | Rayna II | Spot 1 2017 | 1 | |  |  |
|  |  | Spot 2 2017 |  | | 1 |  |
|  |  | Spot 3 2017 |  | |  | 1 |
|  | Kalna I | Spot 1 2017 | 1 | |  |  |
|  |  | Spot 2 2017 |  | | 1 |  |
|  |  | Spot 3 2017 |  | |  | 1 |
